# The genomic basis of evolutionary differentiation among honey bees

**DOI:** 10.1101/gr.272310.120

**Published:** 2021-07

**Authors:** Bertrand Fouks, Philipp Brand, Hung N. Nguyen, Jacob Herman, Francisco Camara, Daniel Ence, Darren E. Hagen, Katharina J. Hoff, Stefanie Nachweide, Lars Romoth, Kimberly K.O. Walden, Roderic Guigo, Mario Stanke, Giuseppe Narzisi, Mark Yandell, Hugh M. Robertson, Nikolaus Koeniger, Panuwan Chantawannakul, Michael C. Schatz, Kim C. Worley, Gene E. Robinson, Christine G. Elsik, Olav Rueppell

**Affiliations:** 1 Department of Biology, University of North Carolina at Greensboro, Greensboro, North Carolina 27403, USA;; 2 Institute for Evolution and Biodiversity, Molecular Evolution and Bioinformatics, Westfälische Wilhelms-Universität, 48149 Münster, Germany;; 3 Department of Evolution and Ecology, Center for Population Biology, University of California, Davis, Davis, California 95161, USA;; 4 Laboratory of Neurophysiology and Behavior, The Rockefeller University, New York, New York 10065, USA;; 5 MU Institute for Data Science and Informatics, University of Missouri, Columbia, Missouri 65211, USA;; 6 Centre for Genomic Regulation (CRG), The Barcelona Institute of Science and Technology, 08036 Barcelona, Spain;; 7 School of Forest Resources and Conservation, University of Florida, Gainesville, Florida 32611, USA;; 8 Department of Human Genetics, University of Utah, Salt Lake City, Utah 84112, USA;; 9 Department of Animal and Food Sciences, Oklahoma State University, Stillwater, Oklahoma 74078, USA;; 10 University of Greifswald, Institute for Mathematics and Computer Science, Bioinformatics Group, 17489 Greifswald, Germany;; 11 University of Greifswald, Center for Functional Genomics of Microbes, 17489 Greifswald, Germany;; 12 Department of Entomology, University of Illinois at Urbana-Champaign, Urbana, Illinois 61801, USA;; 13 Universitat Pompeu Fabra (UPF), 08002 Barcelona, Spain;; 14 New York Genome Center, New York, New York 10013, USA;; 15 Utah Center for Genetic Discovery, University of Utah, Salt Lake City, Utah 84112, USA;; 16 Department of Behavioral Physiology and Sociobiology (Zoology II), University of Würzburg, 97074 Würzburg, Germany;; 17 Environmental Science Research Center (ESRC) and Department of Biology, Faculty of Science, Chiang Mai University, Chiang Mai 50200, Thailand;; 18 Departments of Computer Science and Biology, Johns Hopkins University, Baltimore, Maryland 21218, USA;; 19 Department of Molecular and Human Genetics, Human Genome Sequencing Center, Baylor College of Medicine, Houston, Texas 77030, USA;; 20 Carl R. Woese Institute for Genomic Biology, University of Illinois at Urbana-Champaign, Urbana, Illinois 61801, USA;; 21 Neuroscience Program, University of Illinois at Urbana-Champaign, Urbana, Illinois 61801, USA;; 22 Division of Animal Sciences, University of Missouri, Columbia, Missouri 65211, USA;; 23 Division of Plant Sciences, University of Missouri, Columbia, Missouri 65211, USA;; 24 Department of Biological Sciences, University of Alberta, Edmonton, Alberta T6G 2E9, Canada

## Abstract

In contrast to the western honey bee, *Apis mellifera*, other honey bee species have been largely neglected despite their importance and diversity. The genetic basis of the evolutionary diversification of honey bees remains largely unknown. Here, we provide a genome-wide comparison of three honey bee species, each representing one of the three subgenera of honey bees, namely the dwarf (*Apis florea*), giant (*A. dorsata*), and cavity-nesting (*A. mellifera*) honey bees with bumblebees as an outgroup. Our analyses resolve the phylogeny of honey bees with the dwarf honey bees diverging first. We find that evolution of increased eusocial complexity in *Apis* proceeds via increases in the complexity of gene regulation, which is in agreement with previous studies. However, this process seems to be related to pathways other than transcriptional control. Positive selection patterns across *Apis* reveal a trade-off between maintaining genome stability and generating genetic diversity, with a rapidly evolving piRNA pathway leading to genomes depleted of transposable elements, and a rapidly evolving DNA repair pathway associated with high recombination rates in all *Apis* species. Diversification within *Apis* is accompanied by positive selection in several genes whose putative functions present candidate mechanisms for lineage-specific adaptations, such as migration, immunity, and nesting behavior.

How genomes diverge to give rise to organismal diversity remains one of the most fundamental questions in biology. Comparative functional genomics has drastically expanded our knowledge on the relative contributions of genetic novelty and co-option (Jasper et al. 2015; Warner et al. 2019), structural and regulatory innovation (Deplancke et al. 2016), as well as *cis*- and *trans*-regulation of gene expression (Green et al. 2019) to phenotypic diversification. As a consequence, the genotype–phenotype map is being elucidated at ever-increasing detail (Zhou et al. 2020). In addition to broad-scale macroevolutionary studies, taxon-specific comparative genomics is generating novel insights, particularly with respect to structural genome evolution (Figueiró et al. 2017; Chavez et al. 2019; Sun et al. 2021).

The evolution of complex insect societies represents one of the major evolutionary transitions (Maynard Smith and Szathmáry 1995). Genomic signatures of this transition share few commonalities across taxa, except for an increase in gene regulatory capacity (Gadau et al. 2012; Simola et al. 2013; Terrapon et al. 2014; Kapheim et al. 2015; Harpur et al. 2017; Harrison et al. 2018). In contrast to the major focus on studying the genomic bases of the origin of sociality and associated traits, the maintenance and diversification of social traits has received limited attention (Simola et al. 2013; Jasper et al. 2015; Araujo and Arias 2021; Sun et al. 2021).

Here, we use a comparative, lineage-specific approach to identify genetic loci associated with evolutionary adaptations underlying the organization of complex insect societies in the eusocial honey bee genus *Apis*. Because of its scientific and practical importance, the western honey bee *Apis mellifera* (L.) was among the first metazoans with a completed genome project (Weinstock et al. 2006). It has since served as a model for genomic studies of adaptation (Wallberg et al. 2014), invasion (Calfee et al. 2020), and social traits such as caste differentiation (Chen et al. 2012), division of labor (Smith et al. 2008), and other social behaviors (Zayed and Robinson 2012).

In addition to the cavity-nesting *A. mellifera* and closely related species, the genus *Apis* contains two other lineages: the dwarf honey bees and giant honey bees (Raffiudin and Crozier 2007). Although their evolutionary origins are not clear (Kotthoff et al. 2013), all species share a social lifestyle in complex societies with thousands of workers and a single, polyandrous queen and nest in vertical wax comb to store food and raise brood (Oldroyd and Wongsiri 2006). However, the three subgenera show pronounced differences in body size, colony size, mating behavior, caste divergence, nesting habits, thermoregulatory ability, recruitment dances, and defensive and migratory behaviors (Dyer and Seeley 1991; Oldroyd and Wongsiri 2006; Koeniger et al. 2010; Hepburn and Radloff 2011; Rueppell et al. 2011b).

The genetic architecture underlying the diversification of the *Apis* lineages remains largely unknown. Intra-specific studies have addressed the genetic basis of some key social traits, such as worker ovary size and caste differentiation (Cardoen et al. 2011; Graham et al. 2011; Chen et al. 2012), dance language (Johnson et al. 2002), and defensive behavior (Hunt et al. 2007; Alaux et al. 2009) in *A. mellifera*. However, it is unclear to what extent the identified genetic mechanisms involved in intra-specific variation can explain the inter-specific differentiation among *Apis* species (Dieckmann et al. 2004). Broad comparisons in *Apis* (Sarma et al. 2007, 2009) have been hampered by the lack of available genomic resources in species other than *A. mellifera* (Weinstock et al. 2006; Elsik et al. 2014) and the closely related *A. cerana* (Park et al. 2015), although the genome of *A. dorsata* has recently also been published (Oppenheim et al. 2020) and targeted analyses have helped to resolve particular gene families (Helbing et al. 2017).

Here, we present a comprehensive analysis of the molecular evolution of protein-coding genes across *Apis* based on homologous gene sets derived from genomes of all three major honey bee lineages. At the genome level, we reconstruct the phylogenetic relationships among the *Apis* lineages and identify key targets of positive selection associated with social complexity, ecological specialization, and chemosensation, elucidating the genomic basis of evolutionary diversification within honey bees.

## Results

### Honey bee genomes and phylogenetic inference

We identified all single-copy orthologs between the western honey bee *Apis mellifera*, the dwarf honey bee *A. florea*, and the giant honey bee *A. dorsata*, with bumblebees as an outgroup. Our analysis included the published genomes of *A. mellifera* (Elsik et al. 2014) and *Bombus impatiens* and *B. terrestris* (Sadd et al. 2015). In addition, we sequenced, assembled, and annotated the genomes of *A. florea* and *A. dorsata*. This produced two high-quality genome assemblies of similar length and GC content (*A. dorsata*: 230 Mb, N50: 732 kb, GC: 32.5%; *A. florea*: 229 Mb, N50: 2.86 Mb, GC: 34.9%) but different contiguity (*A. dorsata*: size of scaffolds: 200 bp–3.6 Mb, total count: 4040; *A. florea*: size of scaffolds: 500 bp–9.6 Mb, total count: 6983), likely explained by differences in repetitive sequences (*A. dorsata*: 17.5%, 40.4 Mb; *A. florea*: 14.3%, 32.9 Mb). Although a newer assembly for *A. mellifera* has been published since our analysis (Wallberg et al. 2019) and our sequencing and assembly strategies for *A. florea* and *A. dorsata* have been replaced by more modern approaches (Phillippy 2017), the generated data sets proved to be informative and appropriate for our subsequent analyses: A high level of gene completeness (*A. dorsata*: 93.7%, *A. florea*: 91.9%) was confirmed by a BUSCO analysis (Simão et al. 2015) with the Hymenoptera lineage data set.

**Figure 1.**
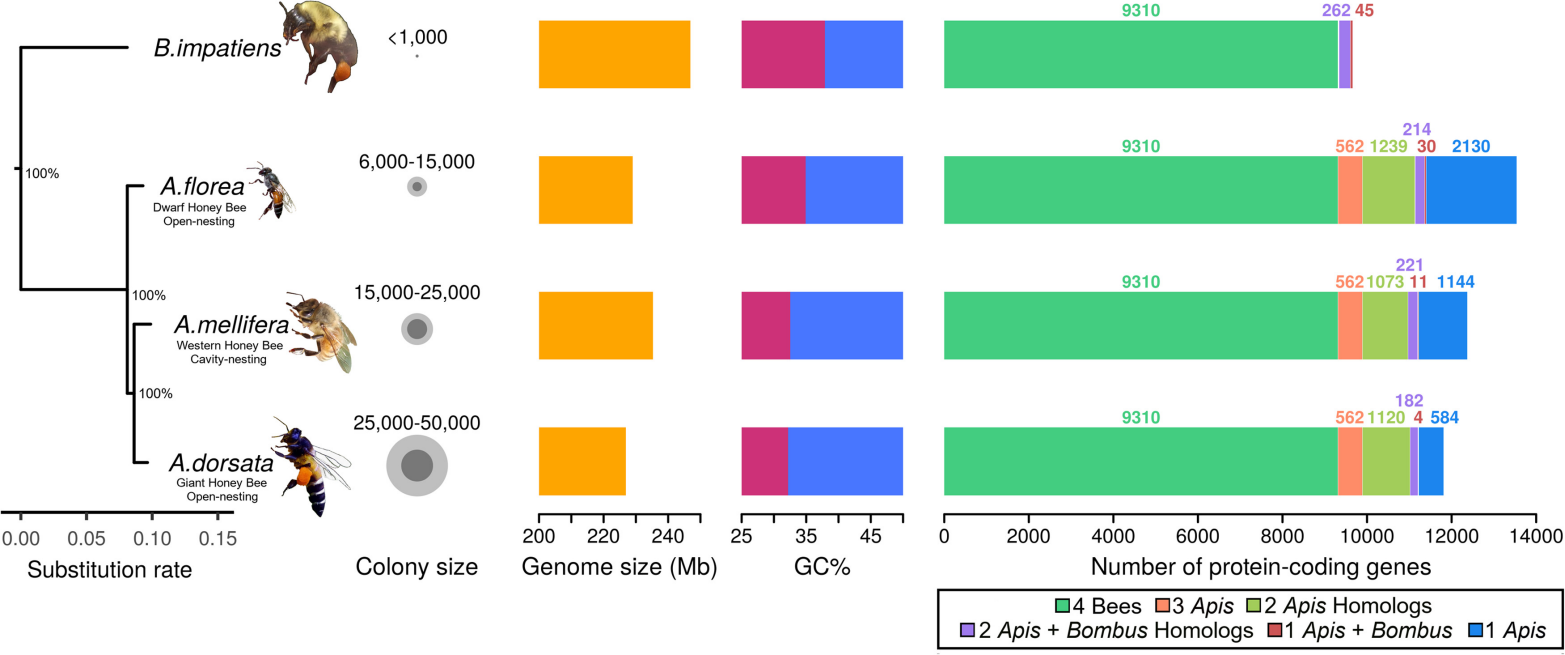
Phylogenetic, genomic, and gene content comparisons of three honey bee species. (*Left* to *right*) Maximum likelihood phylogeny built from 9310 concatenated single-copy orthologous proteins from sequenced honeybees and bumblebee outgroup indicated that *A. florea* diverged first from the most recent common ancestor of honey bees (all nodes 100% bootstrap supported). *A. florea* represents the dwarf honey bees, and *A. mellifera* and *A. dorsata* represent the cavity nesters and the giant honey bees, respectively. Tree visualization was performed using ggtree (Yu 2020). Circles represent colony size ranges with dark gray indicating the lowest and light gray the highest colony size; the yellow bars depict the genome size of each species, and the red/blue bars correspond to the average GC content of the genome of each species. Average genome GC content decreases with increasing colony size. The *rightmost horizontal* bar plots show total gene counts for each species partitioned according to their orthology profiles*. A. florea* possessed the greatest number of lineage-specific genes followed by *A. mellifera*.

The gene sets for comparison across species (Methods) were of similar size among all bees (Fig. 1). A total of 3858 genes were present in only a single species (2130 in *A. florea*, 584 in *A. dorsata*, and 1144 in *A. mellifera*) and thus were categorized as lineage specific. Among the 1506 genes identified as homologs in only two species, 570 were shared between *A. mellifera* and *A. dorsata* (570), more than either species with *A. florea* (386 and 550, respectively). Among all species, 15,182 genes were shared with 9310 belonging to single-copy ortholog groups (Fig. 1). The concatenated single-copy orthologs resulted in an alignment of 4,680,591 amino acids, which we used to resolve the relationships among the three honey bee lineages. We recovered a highly supported phylogeny of *Apis* with the dwarf honey bees as an outgroup to the other two lineages (Fig. 1), agreeing with previous work (Raffiudin and Crozier 2007).

### Genome-wide patterns of positive selection

To identify positive selection that acted on protein-coding genes during the evolution of honey bees, we used the adaptive branch-site random effects likelihood (aBRSEL) method in Hyphy (Smith et al. 2015; Kosakovsky Pond et al. 2019) on 8115 single-copy orthogroups (Methods). We identified 149 single-copy orthogroups (1.85%) with signals of positive selection in at least one of the four branches at a 10% false discovery rate (FDR). Patterns of positive selection were equally distributed among the three honey bee species lineages with a proportion of 0.49%–0.60% of all orthogroups tested (Supplemental Tables S1, S2). The basal *Apis* branch, however, was under positive selection in only 0.27% of orthogroups, representing a significantly lower proportion in comparison to the three species branches (χ^2^ test: χ^2^ = 10.48, d.o.f. = 3, *P* = 0.0149). This result was not caused by reduced power associated with short branches (Anisimova and Yang 2007) because the *Apis* branch had an overall increased branch length (mean branch length [± standard error] of *Apis*: 0.37 ± 0.02, *A. mellifera*: 0.06 ± 0.0005, *A. florea*: 0.05 ± 0.0004, *A. dorsata*: 0.04 ± 0.0003; Kruskal–Wallis test: χ^2^ = 3280, d.o.f. = 3, *P* < 2.2 × 10^−16^) and orthogroup test scores were positively correlated with the length of the tested branches (log-likelihood ratio; Spearman's correlation ρ = 0.20, *P* < 2.2 × 10^−16^).

Next, we categorized each orthogroup by its homology with genes of known function in *A. mellifera*, to test whether the identified patterns of positive selection correlated with known functions. Of the 8115 orthogroups included in the analysis, 6719 (82.8%) could be categorized this way, whereas the function of 1396 (17.2%) remained unknown. The proportion of genes with known (83.1%) and unknown (16.9%) function under positive selection did not differ from the overall distribution (χ^2^ test: χ^2^ < 0.01, d.o.f. = 1, *P* = 1). However, genes with unknown function had a significantly higher median evolutionary rate ratio (*d*_N_/*d*_S(known function)_ = 0.077, *d*_N_/*d*_S(unknown function)_ = 0.157; Wilcoxon rank-sum test: W = 5.4 × 10^7^, *P* < 2.2 × 10^−16^) compared to those with a known function. Although this result is not surprising because genes with higher divergence rates are more difficult to annotate based on homology with genes of known function, it does emphasize the significance of studying genes of unknown function.

Most of the significant gene families were found to be positively selected in a single branch, although the following five were found to be positively selected in two branches: (1) *muscle myosin heavy chain*, which is involved in muscle contraction (Holmes 2004; Odronitz and Kollmar 2008), was under positive selection in both *A. dorsata* and *A. florea*; (2) *four and a half LIM domains protein 2*, involved in heart physiology and muscle formation (Johannessen et al. 2006), was under positive selection in both *A. dorsata* and *mellifera*; (3) *serine-rich adhesin for platelets*, which plays a role in cell adhesion (Sanchez et al. 2010), was positively selected in the *Apis* branch and in *A. florea*; and (4) *alpha-glucosidase 2* (*AmGCS2α*), which is involved in glucose metabolism, and (5) one additional orthogroup of unknown function were positively selected in both the *Apis* branch and *A. mellifera*. In the three species branches, as well as the ancestral *Apis* branch, several positively selected genes were identified with a function in the regulation of gene expression, cell signaling, and neural processes, as well as with an association with resistance against pathogens and xenobiotics (Supplemental Tables S1, S2).

### Tests of functional category enrichment

**Figure 2.**
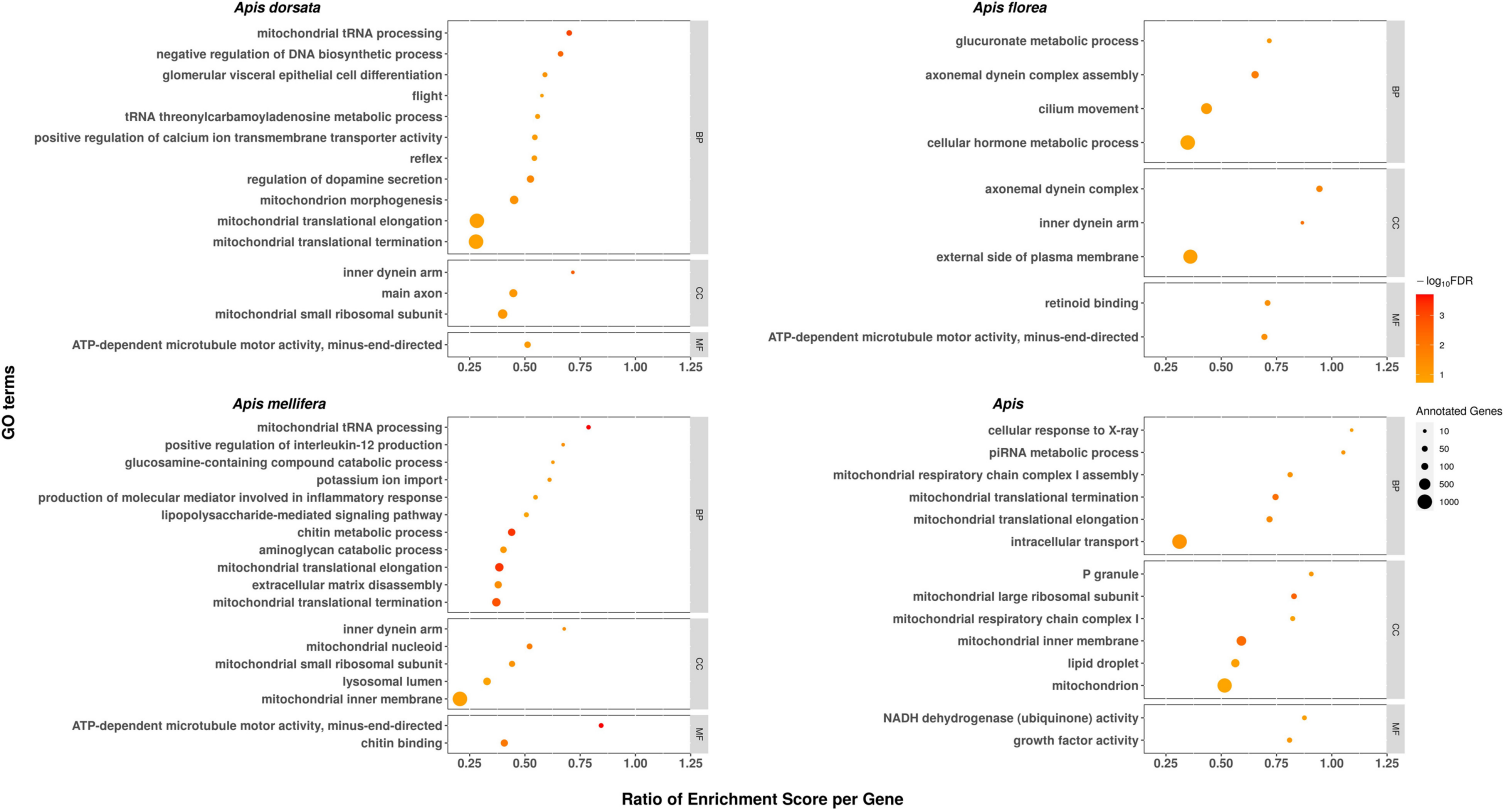
Functional categories enriched with genes under positive selection in each honey bee species and their most recent common ancestor. GO terms enriched in positively selected genes are depicted as spheres representing the number of annotated genes (sphere size) and the −log_10_ of their FDR (color intensity). GO enrichment scores, normalized by the number of annotated genes, are indicated by the *x*-axis. Most enriched GO terms with positively selected genes can be interpreted as adaptations to long-distance migration and increased colony size in *A. dorsata*, colony defense in *A. florea*, immunity in *A. mellifera*, and TE silencing and high recombination rates in the basal *Apis* lineage. (BP) Biological process; (CC) cellular component; (MF) molecular function.

**Figure 3.**
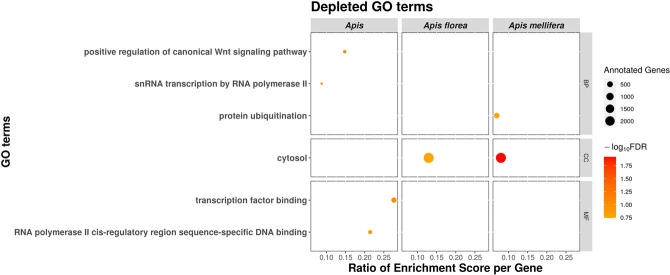
Functional categories depleted of genes under positive selection in each honey bee species and their most recent common ancestor. Spheres indicate GO terms depleted of positively selected genes, for which size represents the number of annotated genes and color intensity the significance (−log_10_ of their FDR). The *x*-axis represents the normalized GO enrichment score divided by the number of annotated genes. Most of the GO terms depleted in genes under positive selection are found in the basal *Apis* branch and relate to transcription functions. No depleted GO term was found in *A. dorsata*. (BP) Biological process; (CC) cellular component; (MF) molecular function.

To identify whether positive selection across the honey bee species quantitatively relates to particular functions, we classified genes based on their Gene Ontology (GO) annotation from *A. mellifera* orthologs. Using SUMSTAT (Roux et al. 2014) with the topGO R package (Alexa et al. 2006) to test for gene set enrichment, we identified 51 significant functional categories, of which 45 were enriched and six depleted in genes under positive selection at 20% FDR. Most functional categories enriched with positively selected genes were unique for each branch, with the exception of “ATP-dependent microtubule motor activity,” which was shared among the three *Apis* species and “mitochondrial translation-related functions,” which was enriched in all branches but *A. florea* (Fig. 2). In addition, *A. dorsata* and *A. mellifera* shared similar functional categories involved in cellular ion exchange (Supplemental Table S3). GO terms depleted of positively selected genes were mostly found in the *Apis* branch and were linked to the regulation of transcription (Fig. 3).

The *Apis* branch revealed 14 enriched GO categories including the “piRNA metabolic process” and “cellular response to X-ray.” The former could relate to the particularly low TE content of honey bees (Petersen et al. 2019) because piRNAs silence transposable elements (Ernst et al. 2017), but the latter might explain the honey bees’ high genomic recombination rates (Rueppell et al. 2016) owing to its link to DNA double-strand breaks (DSB) that are required to initiate recombination (Aguilera and Gómez-González 2008). GO categories enriched in *A. florea* included “hormone and glucuronate metabolism,” and “retinal proteins.” The GO categories “glomerular visceral epithelial cell differentiation,” “dopamine metabolism,” “flight,” and “negative regulation of DNA biosynthesis” were enriched for positive selection in *A. dorsata*. The *A. mellifera* branch was enriched in “chitin metabolism” and “inflammatory response.”

### Overlap analyses

A comparison of genes we identified as positively selected with published lists of genes of functional significance in *Apis* identified numerous overlapping genes (Supplemental Table S4) but did not reveal any quantitatively significant overlap. None of our four lists (*Apis* branch, *A. florea* branch, *A. dorsata* branch, and *A. mellifera* branch) showed significantly more overlap than expected by chance with inter-specific differences in brain gene expression (Sarma et al. 2007). There was also no significant overlap with functional gene lists identified by intra-specific studies, such as selected genes within *A. mellifera* (Wallberg et al. 2014), genes involved in *A. mellifera* caste determination (Chen et al. 2012), worker reproduction (Cardoen et al. 2011), worker behavioral ontogeny (Whitfield et al. 2006; Khamis et al. 2015), and queen–worker brain differences (Grozinger et al. 2007). The largest overlap (*P* = 0.0012) was found between genes selected in the *A. mellifera* branch and genes in the midgut that were up-regulated in *A. mellifera* foragers compared to nurses (Jasper et al. 2015), but correcting for the 72 independent comparisons made to this particular data set alone rendered the overlap nonsignificant.

The positively selected genes were also compared to positional candidates in the confidence intervals of published intra-specific quantitative trait loci for the pollen hoarding syndrome, specifically foraging behavior (pln1–4) and ovary size (wos1–5) (Hunt et al. 2007; Graham et al. 2011; Rueppell et al. 2011a). Nine positively selected genes were located in these genome regions. Five of these genes showed evidence of selection in the *A. dorsata* branch and none in the *Apis* branch. Known functions of the genes were diverse with a bias toward functions in the nervous system (Table 1).

**Table 1.**
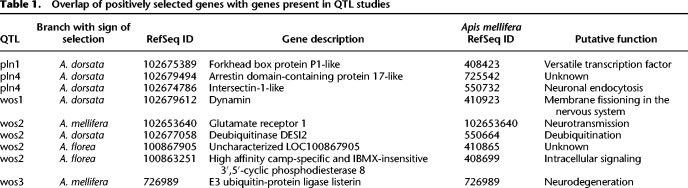
Overlap of positively selected genes with genes present in QTL studies

### Lineage-specific genes

Lineage-specific genes have received increased attention owing to their potential role in lineage- or species-specific trait evolution (Simola et al. 2013; Jasper et al. 2015). To understand the role of lineage-specific genes in the diversification of honey bees, we performed a gene set enrichment analysis by comparing GO term annotations of the lineage-specific genes (Fig. 1) to our orthogroups. The majority of lineage-specific genes (1994 in *A. florea* [92.2%], 560 in *A. dorsata* [95.2%], and 1218 in *A. mellifera* [91.5%]) could not be categorized into a functional group nor into previously characterized protein families (Supplemental Table S5). Accordingly, the GO analysis revealed only a few enriched terms for *A. florea* at 20% FDR, including “carbohydrate metabolic process,” “hydrolase activity, hydrolyzing O-glycosyl compounds,” and “DNA integration” (Supplemental Table S5). Although not significantly enriched in the GO term analysis, the *A. dorsata* genome contained two lineage-specific genes related to vision, *gelsolin-like* and *calphotin-like*, and the *A. mellifera* genome also revealed several lineage-specific genes of interest (Supplemental Table S5).

### Chemosensory gene evolution

Chemosensory diversification is important for insect evolution (McBride et al. 2014; Brand et al. 2020) but automated annotation of chemosensory genes remains problematic. Thus, we manually annotated and analyzed five chemosensory gene families involved in olfaction and gustation: odorant binding proteins (OBPs), chemosensory proteins (CSPs), odorant receptors (ORs), gustatory receptors (GRs), and ionotropic receptors (IRs) (Sánchez-Gracia et al. 2009; Croset et al. 2010).

**Figure 4.**
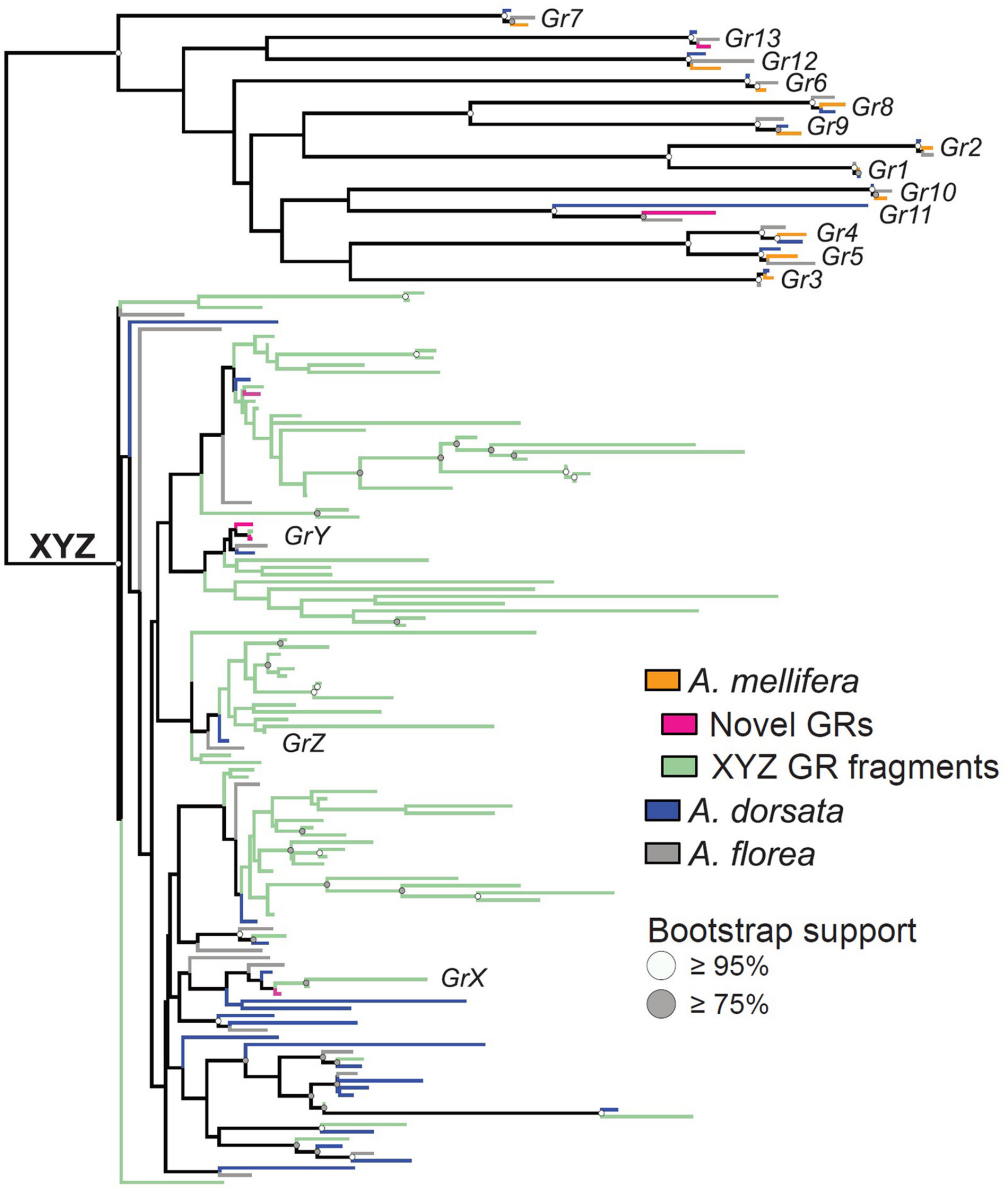
Gustatory receptor (GR) gene family phylogeny including newly annotated genes of three honey bee species. The maximum likelihood tree contained two clades, one including a single ortholog of all putatively functional GRs previously described in *A. mellifera* (in orange) in each species (blue: *A. dorsata*; gray: *A. florea*), and the XYZ clade (supported with 99% bootstrap support) previously thought to be entirely pseudogenized (Robertson and Wanner 2006; Sadd et al. 2015). Five newly identified full-length GRs for *A. mellifera* are highlighted in pink, some of which are among the newly identified XYZ GRs (four in *A. mellifera*, 15 in *A. florea*, and 19 in *A. dorsata*). All GR groupings outside the XYZ clade have high bootstrap support (for exact support values, see Supplemental Fig. S2), highlighting the conservation of GR gene number in this group across *Apis*. In addition to >50 small fragments with homology to GRs (light green, only *A. mellifera* fragments shown), we newly identified a number of full-length genes in the XYZ clade, all of which are supported by gene expression data in *A. mellifera*. The fragments are included here to represent all of our results, although the GR phylogeny is much clearer without them (Supplemental Fig. S2). With 16–26 putatively functional GRs per species, honey bees are similar to other corbiculate bees (Brand and Ramírez 2017), suggesting that the sense of taste in honey bees is more sophisticated than previously thought.

The number of chemosensory genes in *A. dorsata* and *A. florea* (Supplemental Table S6) was similar to the previously described gene sets in *A. mellifera* for all chemosensory gene families (Robertson and Wanner 2006; Karpe et al. 2016; Brand and Ramírez 2017), with a large number of 1:1:1 orthologous genes between the three species (from 66% in ORs to 100% in CSPs and IRs). Additionally, we found conservation of genes, such as the 9-ODA receptor gene *OR11*, across species. Although we did not detect any variation in CSPs and IRs across the honey bees, OBPs, ORs, and GRs varied in the number of genes, revealing gains and losses (Fig. 4; Supplemental Figs. S1, S2). The most variable clades in all three of these gene families, previously identified as specific to honey bees in comparison to other corbiculate bees (Brand and Ramírez 2017), were similar in numbers for all three species analyzed but revealed complex phylogenetic relationships, including the OR 9-exon subfamily.

In addition to these patterns shared among gene families, we found that the number of GRs in the newly annotated *A. florea* and *A. dorsata* genomes differed substantially from *A. mellifera*. Previous annotations of the *A. mellifera* genome reported a total of 15 GR genes including 11 functional and four pseudogenized copies (Robertson and Wanner 2006; Smith et al. 2011). In addition to single copies for each of the functional GRs known from *A. mellifera*, we identified 19 and 15 GRs in *A. dorsata* and *A. florea*, respectively (Fig. 4). Of these, eight and two were likely pseudogenes, respectively, and all of these GRs formed a monophyletic clade with the three previously described X, Y, and Z *A. mellifera* pseudogenes (Fig. 4). Several of the XYZ-homologous GRs showed 1:1 homology between *A. dorsata* and *A. florea*, as well as the *A. mellifera* pseudogenes. A reannotation of the *A. mellifera* GR gene family, including the previously reported >50 fragmented GR pseudogenes (Robertson and Wanner 2006), reconstructed all known functional GRs and 88 additional sequences with homology with the X, Y, and Z GR pseudogenes. Six of 11 GRs with a length of at least 300 amino acids contained premature stop codons, whereas the other five represent new, potentially functional GRs.

To validate potential functionality of the newly described GRs, we visualized gene models along with RNA-seq tracks in the *A. mellifera* Apollo browser (Dunn et al. 2019) available at the Hymenoptera Genome Database (Elsik et al. 2016). Four of the GR gene models were supported by RNA-seq reads spanning predicted exon–intron boundaries, indicating they are actively transcribed and thus functional receptors. The only novel full-length GR without expression support was highly similar to *GR13*, which was also present in the genomes of *A. dorsata* and *A. florea* and has known orthologs in several other corbiculate bees (Brand and Ramírez 2017), suggesting it is a conserved functional GR as well. Several of the smaller fragments were also supported by expression data, suggesting that they might be part of coding genes that are not well assembled. Indeed, all but one of the newly identified GR sequences were located on small scaffolds not assigned to linkage groups (“Un”-scaffolds), and gene models were often truncated at the end of a scaffold. Accordingly, it is likely that the additional five GRs we identified for *A. mellifera* are an underestimation of the real number of honey bee–specific GRs in the XYZ subfamily (Brand and Ramírez 2017).

## Discussion

Fine-scale comparative genomic analyses lead to a better understanding of the molecular basis of species diversification and increased resolution of genomic feature evolution. Our genome-wide analysis reveals increased positive selection pressure during the diversification of the three honey bee lineages after the divergence of *Apis* from its most recent common ancestor with *Bombus*. Our results parallel previous analyses that indicate accelerated evolution during the diversification of species within a family (Nevado et al. 2016; Tollis et al. 2018; Vianna et al. 2020), suggesting a common evolutionary pattern. We also find evidence for selection for sequence changes in existing protein-coding regions and evolutionary turnover of genes, similar to a genomic study of the radiation of closely related bumble bees (Sun et al. 2021). These two sources of evolutionary change may be important in bee social evolution in addition to regulatory diversification (Kapheim et al. 2015). Practically, rapid evolutionary divergence may not be easy to distinguish from evolution of novel genes, unless sufficient similarity remains to distinguish orthologs from paralogs as in our manual *Apis* chemoreceptor analyses. We believe that our extensive search for taxonomically restricted genes resulted in unrealistically high estimates of novel genes because the majority of these genes have only support from one prediction method. However, the findings suggest the existence of at least some additional species-specific genes within *Apis* that deserve further study.

We did not identify significant overlap between the genes found to be positively selected among species and genes that determine intra-specific variation in key traits of honey bees, which we predicted based on the hypothesis that phenotypic plasticity is a main driver of *Apis* diversification (West-Eberhard 2003; Kapheim et al. 2020). In contrast to the stark phenotypic differences of honey bees to their closest contemporary relatives, relatively few genes were identified as positively selected in the shared evolution of all honey bees (basal *Apis* branch) compared to the number of positively selected genes detected across branches within *Apis* (species branches). Although we lack a comprehensive explanation for the relatively low number of positively selected genes, it is plausible that evolution at this stage was more strongly driven by gene regulatory changes (Kapheim et al. 2015) or the appearance of *Apis*-specific genes.

In additional to the computational prediction of additional genes, our manual analysis corrected previous results of low numbers of GR genes in honey bees (11 GRs) (Robertson and Wanner 2006): We were able to identify 22, 26, and 16 complete GR genes in *A. dorsata*, *A. florea*, and *A. mellifera*, respectively, aided by an updated genome assembly for *A. mellifera* (Elsik et al. 2014). This increase of full-length GRs in *A. mellifera* by almost 50% is presumably still an underestimate owing to low quality sequence assembly of the respective parts of the genome. Thus, the sense of taste in honey bees may be more sophisticated than previously thought (Wright et al. 2010). Furthermore, the XYZ subfamily, which is only found in *Apis* (although one instance has been reported from *Bombus terrestris*) (Sadd et al. 2015), revealed complex evolutionary dynamics suggesting an evolutionary history of gustatory functions specific to honey bees. Together, this makes the XYZ subfamily an interesting target to understand the evolution of chemosensory capabilities in honey bees.

### The evolution of *Apis* supports previous studies on the molecular basis of increased social complexity

The rise of eusociality in insects has been linked with an increased capacity of gene regulation and the rapid evolution of chemoreceptors, despite the small number of fast-evolving genes shared among eusocial insects (Woodard et al. 2007; Simola et al. 2013; Terrapon et al. 2014; Kapheim et al. 2015, 2020; Harrison et al. 2018).

Although our analyses support the importance of chemosensation, we found that the divergence of the *Apis* ancestor from the most recent common ancestor with *Bombus* was accompanied by a depletion of positively selected genes from functional categories related to transcription, such as “transcription factor binding.” The major evolutionary transition to eusociality was not captured in our contrast between *Bombus* and *Apis* and our results may thus reflect a subsequent conservation of gene regulatory mechanisms that consolidate and stabilize the progress of a rapid transition to sociality. Subsequent gene regulatory changes in the evolution of *Apis* may have been achieved by more specific mechanisms: Genes involved in growth factor activity, a major pathway of the regulation of gene expression, were fast evolving in the ancestor of all *Apis* species. The rapid evolution of piRNA metabolism in honey bees might also be linked to the regulation of gene expression in *Apis*, as it regulates gene expression and epigenetic effects in *Drosophila* (Weick and Miska 2014; Glastad et al. 2019) and piRNAs target regions antisense of protein-coding genes in honey bees, suggesting that they could control transcription (Wang et al. 2017).

Chemosensory gene evolution has been hypothesized to be important during the evolution of eusociality (Harrison et al. 2018). The 9-exon OR gene family has been hypothesized to be important in social communication in Hymenoptera, owing to a role of 9-exon ORs in the detection of CHCs in ants (Smith et al. 2011; McKenzie et al. 2016; Pask et al. 2017; Slone et al. 2017). Our results show that the OR 9-exon subfamily evolves rapidly between the three *Apis* species, which occurs also more widely (Sadd et al. 2015; Brand and Ramírez 2017). In contrast, sex pheromone receptor genes (*OR11*, *OR10*, *OR18*, and *OR170*) were highly conserved. Moreover, we found that the expansion of OBPs is not specific to *A. mellifera* (Brand and Ramírez 2017) but most likely occurred in the common ancestor of *Apis* species, pointing to a role in chemosensory behaviors unique to honey bees.

### *Apis* evolution reveals an evolutionary trade-off between genome stability and variability

Although genome stability is vital for organisms and crucial for maintenance of optimally adapted phenotypes, it restricts genetic diversity, which is essential for evolutionary and physiological processes, particularly in eusocial insects (Mattila and Seeley 2007; Seeley and Tarpy 2007; Kent et al. 2012). The resulting trade-off between genome stability and diversity was reflected in our findings that TE silencing and DSB repair pathways in the *Apis* lineage were positively selected. The honey bee genomes are depleted of TEs (Elsik et al. 2014; Park et al. 2015) and we found that the regulation of one of the major mechanisms to prevent TE spread within a genome, piRNAs (Brennecke et al. 2007; Ernst et al. 2017), was positively selected in *Apis*. The enrichment of the piRNA regulatory pathway, as well as the GO term “P granule cellular component” (Lim and Kai 2007), among positively selected genes in the *Apis* lineage suggests that positive selection can act on piRNAs over evolutionary time to limit the spread of TEs despite consistently high rates of recombination (Rueppell et al. 2016).

The high recombination rates of all *Apis* species studied so far, ranging from 20 to 25 cM/Mb (Hunt and Page 1995; Meznar et al. 2010; Ross et al. 2015; Rueppell et al. 2016), may increase genetic diversity and facilitate evolutionary novelties (Kent et al. 2012). The enrichment of rapidly evolving genes associated with the cellular response to X-rays in the *Apis* ancestor indicates a corresponding adaptation to double-strand breaks (DSBs) of DNA (Rothkamm and Löbrich 2003). It is unclear whether this selective signature should be interpreted as a cause or consequence of the high recombination rates, but mutations in genes involved in DSB repair can lead to higher homologous recombination rates (Aguilera and Gómez-González 2008). The accelerated molecular evolution of DSB repair genes may thus have enabled the high meiotic recombination rates of honey bees, with potential effects on genome evolution and diversity (Kent et al. 2012).

The continuous oogenesis of Hymenoptera (Büning 1994) can exacerbate the accumulation of mutations during later-life meiosis (Bromham and Leys 2005; Thomas et al. 2010), particularly in females that produce numerous offspring. The resulting mutational load is particularly severe in mitochondria (Neiman and Taylor 2009). Nuclear genomes can coevolve to compensate the loss of mitochondrial function via the accumulation of deleterious mutations (Hill 2020), resulting in increased evolutionary rates of mitochondrion-destined nuclear genes (Li et al. 2017). Correspondingly, we found positive selection of nuclear genes involved in the mitochondrial translation elongation and termination pathway in the *Apis* lineage and in the *A. mellifera* and *A. dorsata* branches, the two species with the largest colony sizes, suggesting selection for increased efficiency and accuracy of mitochondrial translation (Schneider 2011) in the face of increased mutations with colony size increases. This hypothesis is also compatible with the strong positive selection targeting the negative regulation of DNA biosynthesis and the tRNA threonylcarbamoyladenosine metabolism essential for accurate translation (Yarian et al. 2002) in *A. dorsata*, the honey bee species with the greatest colony size (Oldroyd and Wongsiri 2006). Hence, the molecular evolution of honey bee genomes suggests an evolutionary trade-off between maintaining genome integrity and generating genetic diversity.

### Fine-scale comparative genomics reveals candidates for the evolution of key phenotypic traits

Accordingly with fundamental differences in body size and queen–worker caste divergence among the three *Apis* lineages (Oldroyd and Wongsiri 2006; Rueppell et al. 2011b), we found several positively selected genes predicted to belong to gene families involved in growth and reproductive processes: a *G-protein-coupled receptor* with similarities to the life-history regulator *methuselah* (Delanoue et al. 2016) and the ovary determinant *tudor* (Xie et al. 2019) in the basal *Apis* branch, *pde8* involved in ERK-signaling that has multiple life-history coordinating roles (Brown et al. 2013) in the *A. florea* branch, and the putative growth effectors *short neuropeptide F receptor* (Lee et al. 2008), *farnesol-dehydrogenase* (Mayoral et al. 2009), and *cdk2* (Vidwans and Su 2001) in the giant honey bee lineage.

The evolutionary diversification of nesting behavior into cavity nesting in *A. mellifera* and related species versus open nesting in the other lineages has been highly controversial for decades and has direct ramifications for understanding the evolution of the honey bee dance language (Koeniger 1976; Oldroyd and Wongsiri 2006; Raffiudin and Crozier 2007; Koeniger et al. 2011). Our analysis cannot resolve this controversy but provides some support for a transition from cavity nesting to open nesting within *Apis*: Although no genes or GO terms that could be interpreted as adaptations to open nesting were found to evolve under positive selection in the ancestral *Apis* branch, in *A. florea*, which accurately controls nest temperature despite its open-nesting habit (Oldroyd and Wongsiri 2006), lineage-specific genes were associated with carbohydrate metabolism, a pathway associated with thermoregulation in bees (Woodard et al. 2011).

Although all honey bees migrate, only giant honey bees seasonally migrate over long distances, up to 100–200 km in *A. dorsata* (Oldroyd and Wongsiri 2006). Correspondingly, we found potential molecular signatures of adaptations to long-distance migration in the *A. dorsata* lineage: positive selection in genes linked to “flight” along with large musculature and body size (Dulta and Verma 1987), involved in “mitochondrial morphogenesis” that may affect energy metabolism during migration (Sogl et al. 2000; Li et al. 2018); associated with the renal system (i.e., “glomerular visceral epithelial cell differentiation”) allowing water conservation during migration (Wigglesworth 1932); and “regulation of dopamine secretion,” a pathway involved in migration in locusts (Ma et al. 2011). The adaptation to night foraging in *A. dorsata* enables them to detect objects at lower light intensity than expected by their ommatidium structure (Warrant et al. 1996). This might be explained by two *A. dorsata*-specific genes, homologs of genes involved in phototaxis, *gelsolin-like* (Stocker et al. 1999), and vision, *calphotin-like* (Yang and Ballinger 1994). An enhanced floral scent detection in *A. dorsata* may also be beneficial for night foraging, which is suggested by the lineage-specific duplications and pseudogenization events of *OR151* and *OR152*, important for detection of floral compounds (Claudianos et al. 2014).

The *A. mellifera* branch is mainly associated with positive selection on genes involved in chitin metabolic processes, as previously found to be enriched in positively selected genes in *A. mellifera* and bumble bees (Harpur et al. 2014; Sun et al. 2021). They mostly relate to caste differentiation (Li et al. 2012; Malka et al. 2014; Santos and Hartfelder 2015) and immunity (Harpur and Zayed 2013; Oddie et al. 2018), which may be caused by pathogen pressure in the relative stable and long-lasting nests of cavity-nesting species.

Focusing on the main lineages of the unique honey bee genus, our study identifies positively selected genes that warrant further study. Of particular interest are selected genes with putative molecular functions that may link them to key adaptations and the diversification among *Apis* species. Although the genus *Apis* is small and contains only the three subgeneric lineages included in this study, sequencing other *Apis* species to increase phylogenetic depth may further refine our conclusions about *Apis* evolution and enhance our understanding of genome evolution in dwarf, giant, and cavity-nesting honey bees. Overall, our results provide an evolutionary scenario of an *Apis* ancestor adapted to building a vertical comb, likely in cavities, that allowed for increased colony size.

## Methods

### Specimen collection

Haploid drones collected from a single colony per species were used for *A. florea* and *A. dorsata* genome sequencing. The samples of *A. florea* were collected in 2009 from Chiang Mai, Thailand. The samples of *A. dorsata* were collected in the vicinity of the Agricultural Research Station Tenom (Sabah, Malaysia: 5.4° N, 115.6° E) in March 2007. Samples were preserved in RNAlater and subsequently frozen until total DNA extraction from single individuals.

### Genome sequencing and assembly

Two types of WGS libraries, a fragment library and mate-pair libraries with 8-kb inserts, were used to generate the *Apis florea* genome sequencing data using 454 Titanium technology. The Aflo_1.0 genome assembly was generated by assembling WGS reads using Newbler (2.3-PreRelease-10/19/2009) (Margulies et al. 2005). Reads from each Newbler scaffold were grouped, along with any missing mate-pairs, and reassembled using PHRAP (Bastide and McCombie 2007) in an attempt to close the gaps within Newbler scaffolds.

For *A. dorsata*, four libraries were sequenced on an Illumina GA platform for the assembly: (1) 2 × 125 bp paired-end reads from a 500 bp library; (2) 2 × 125 bp mate-pairs from a 1.2-kbp library; (3) 2 × 125 bp mate-pairs from a 3-kbp library, and (4) 2 × 36 bp mate-pairs from a 5-kbp library. The sequencing reads from all four libraries were first error corrected and trimmed using Quake v0.2.0 (Kelley et al. 2010). Error-corrected reads were then assembled using SOAPdenovo v1.0.5 (Supplemental Methods; Li et al. 2010).

Completeness of the two assemblies was assessed by identifying Benchmarking Universal Single-Copy Orthologs (BUSCOs) using the BUSCO v5beta pipeline in genome mode (Simão et al. 2015). For this analysis, we identified single-copy orthologs based on the hymenoptera_db10.

### Genome annotation

To avoid artifacts stemming from different annotation methods (Supplemental Methods), a combined gene set was created for each species, by adding nonoverlapping genes from different annotation pipelines to a fundamental NCBI RefSeq annotation in the following orders: *A. dorsata*, RefSeq → EVM (Haas et al. 2008) → MAKER (Holt and Yandell 2011) → AUGUSTUS -CGP (Stanke et al. 2008; König et al. 2016; Nachtweide and Stanke 2019); *A. florea*, RefSeq → EVM → AUGUSTUS -CGP → BGI (Kapheim et al. 2015); *A. mellifera*, RefSeq → OGS (Elsik et al. 2014) → AUGUSTUS -CGP. Accuracy of all gene prediction methods were assessed (Supplemental Tables S7, S8) and combined in EVM with different weights (Supplemental Tables S9, S10) based on different sources (Supplemental Tables S11, S12), resulting in 12,172 genes for *A. dorsata* (Supplemental Table S13) and 14,393 for *A. florea* (Supplemental Table S14).

Exonerate protein2genome (Slater and Birney 2005) was used to align protein sequences from each species to the genome assemblies of the other two species (*A. mellifera*: NCBI BioProject [https://www.ncbi.nlm.nih.gov/bioproject/] PRJNA10625 and *Bombus impatiens*: BioProject PRJNA61101 and *B. terrestris* BioProject PRJNA45869). For each species, a new gene model was created wherever there was a protein alignment that did not overlap with an existing gene model. At each new gene locus with more than one alternate species alignment, the alignment with the best score was used to generate a single protein-coding gene model, correcting any artifactual frameshifts in protein and coding sequences. The protein homolog-based gene models were added to the combined gene sets to create the final gene sets, deemed “comparative gene sets,” used in this study. Although some of the protein homolog-based predictions were not of sufficient quality for evolutionary analysis, including them in the comparative gene sets allowed us to determine more realistic numbers of species-specific genes.

### Gene set annotation

We used InterProScan (Zdobnov and Apweiler 2001) to compare protein sequences to InterPro (Finn et al. 2017) protein domain and other motif databases (Supplemental Methods). InterProScan assigns Gene Ontology (GO) (The Gene Ontology Consortium 2000) terms and pathway IDs from KEGG (Chen et al. 2012), MetaCyc (Caspi et al. 2018), and Reactome (Fabregat et al. 2018) based on protein domain content. We used FASTA (Pearson and Lipman 1988) with an E-value threshold of 1 × 10^−6^ to compute reciprocal alignments between *Apis* comparative proteins and a *Drosophila melanogaster* protein set consisting of the longest protein isoform of each gene (annotation version r6.14). We identified reciprocal best hits (RBH) and transferred GO, KEGG, PANTHER, and REACTOME annotations from the *D. melanogaster* protein to the *Apis* protein for each RBH pair, using the annotation files available at FlyBase (Gramates et al. 2017). Finally, we obtained gene descriptions from NCBI for the RefSeq (O'Leary et al. 2016) gene annotations.

### Ortholog prediction

We created ortholog groups containing one gene from the two newly annotated genomes of *Apis dorsata* and *A. florea* and the existing *A. mellifera* genome (Amel_4.5, under BioProject PRJNA10625). Protein sequences from the three comparative gene sets were combined into one file that was used in an all-by-all protein comparison with FASTA (Pearson and Lipman 1988) using an E-value threshold 0.001 to identify single-copy orthologs (Supplemental Methods). This process resulted in 15,182 families of *Apis* orthologs. Of those, 5310 families were flagged because a translational discrepancy in the NCBI GFF or a frameshift/gap in the Exonerate alignment were indicated. After creating the families of *Apis* orthologs, a *Bombus* protein to serve as an outgroup was identified for each family (Supplemental Methods). In total, 9310 *Apis* ortholog families were assigned a *Bombus* protein.

### Multiple sequence alignment

For each ortholog family, the longest protein isoforms for each species were used in multiple sequence alignment with PRANK (v.150803) (Löytynoja and Goldman 2008), and unreliably aligned residues were masked with GUIDANCE (v2.02) (Penn et al. 2010). A custom Python script (Supplemental Code) was then used to replace protein sequences with coding sequences in the multiple alignments, resulting in 8115 gene families after filtering (Supplemental Methods). The mean length of filtered alignment was 1621 nt (median = 1233 nt), ranging from 303 to 22,830 nt.

### Phylogeny

Gene family phylogenies were built using RAxML (v7.2.9) (Stamatakis 2006) from the amino acid sequences (9310 *Apis* ortholog families). For each ortholog family, ModelGenerator was used to select the best amino acid matrix and substitution model (Keane et al. 2006). The species phylogeny was built from a concatenation of all amino acid alignments with *B. impatiens* data (9275), using RaxML with an estimated amino acid matrix based on our data (GTR) and the CAT model (Rokas 2011).

### Branch-site test for positive selection

The adaptive branch-site random effects model (aBSREL) (Smith et al. 2015) from Hyphy software package (Kosakovsky Pond et al. 2019) was used to detect positive selection experienced by a gene family in a subset of sites in a specific branch of its phylogenetic tree. Because of our low phylogenetic depth, test for positive selection was run only on the *Apis*, *A. mellifera*, *dorsata*, and *florea* branches (all “leaves”). To account for multiple testing (Anisimova and Yang 2007), *P*-values from the successive 32,460 tests were corrected using the false discovery rate (FDR) (Benjamini and Hochberg 1995). Because of our stringent alignment filtering and the multiple testing correction as one series, we set our significant threshold at 10%. We visually checked alignments of positive results and excluded GC-biased gene conversion because our ω estimates were negatively correlated with GC content (Spearman's correlation: S = 6.7 × 10^12^, rho = −0.17, *P* < 2.2 × 10^−16^).

### Overlap analysis

Our lists of selected genes were compared to multiple other studies. The only other available inter-specific study (Sarma et al. 2009) and the following intra-specific studies that have identified gene sets of functional significance for the observed inter-specific differences within *Apis* were selected: genes involved in caste determination (Chen et al. 2012), reproductive phenotypes (Grozinger et al. 2007; Cardoen et al. 2011), and genes involved in local adaptation (Wallberg et al. 2014). In addition, overlap to quantitative trait loci for ovary size (Graham et al. 2011; Rueppell et al. 2011a) and social behavior (Hunt et al. 2007; Rueppell 2009) was evaluated.

### Tests of functional category enrichment

Gene Ontology (GO) (The Gene Ontology Consortium 2000) annotations for our gene families were taken from *A. mellifera*, annotated with GO terms as described above. To identify functional biases, the package topGO version 2.4 (Alexa et al. 2006) of Bioconductor (Gentleman et al. 2004) was used with the full data set (before filtering) of genes containing a GO annotation as reference. Functional biases were detected using Fisher's exact test with the “elim” algorithm of topGO and selected based on FDR < 20% (Supplemental Methods). Gene Ontology categories mapped to fewer than 10 genes were discarded. To identify functional categories enriched with genes under positive selection, the SUMSTAT test was used (Supplemental Methods). We performed bidirectional tests to account for enrichment and depletion for positively selected genes in a gene set. Gene Ontology categories mapped to fewer than 10 genes were discarded.

### Lineage-specific genes

We identified genes specific to one or two *Apis* genomes using outputs of the all-by-all FASTA protein comparison and Exonerate protein2genome alignments described above. If all protein isoforms encoded by a particular gene were missing protein or Exonerate alignments to another species, that gene was considered missing in the other species. We excluded genes owing to bacterial contamination (Supplemental Methods). To investigate whether lineage-specific genes of each *Apis* species are associated with features of their biology, their GO annotations were compared to the ortholog families’ data set using Fisher's exact test with the “elim” algorithm of topGO. Gene Ontology categories mapped to fewer than 10 genes were discarded.

### Chemosensory gene family analysis

Annotation and selection analysis of chemosensory gene families followed Brand and Ramírez (2017). In brief, high-quality annotations for *A. mellifera* were used to annotate odorant receptors (Robertson and Wanner 2006), odorant binding proteins (Forêt and Maleszka 2006), chemosensory genes (Forêt et al. 2007), gustatory receptors (Robertson and Wanner 2006), and ionotropic receptors (Croset et al. 2010) using Exonerate (Slater and Birney 2005) coupled with manual curation and, if necessary, correction of gene models for *A. dorsata* and *A. florea*. In addition, we reannotated the OR and GR gene families in *A. mellifera* (Robertson and Wanner 2006) and the OR gene family for *A. florea* (Karpe et al. 2016). The resulting gene models were aligned with MAFFT (Katoh and Standley 2013) and used to reconstruct gene family-specific gene trees with RAxML (Stamatakis 2006) using 20 independent ML searches and 100 bootstrap replicates. Selection analyses were performed with the aBSREL algorithm in HYPHY. ORs were divided into subfamilies as defined in Brand and Ramírez (2017), whereas all other gene families were analyzed as a whole. *P*-values for each independent aBSREL run were corrected for multiple testing using an FDR of 5%.

## Data access

The biological data, sequencing data, assembled genome sequences, and annotations generated in this study have been submitted to the NCBI BioProject database (https://www.ncbi.nlm.nih.gov/bioproject/) under accession numbers PRJNA174631 (*A. dorsata*) and PRJNA45871 (*A. florea*).

## Supplementary Material

Supplemental Material
