# Supplementary material for: The genomic basis of evolutionary differentiation among honey bees
Source: Genome Res. 2021 Jul;31(7):1203–15. doi: 10.1101/gr.272310.120 (PMC8256857; doi:10.1101/gr.272310.120)
Supplement: Supplemental Material [file supp_31_7_1203__DC1.html]

The genomic basis of evolutionary differentiation among honey bees — Supplemental Material 

# The genomic basis of evolutionary differentiation among honey bees

## Supplemental Material

- Supplemental\_Fig\_S1.pdf
- Supplemental\_Fig\_S2.pdf
- Supplemental\_Methods.docx
- Supplemental\_Code.txt
- Supplemental\_Table\_S1.xlsx
- Supplemental\_Table\_S2.xlsx
- Supplemental\_Table\_S7.docx
- Supplemental\_Table\_S8.docx
- Supplemental\_Table\_S9.docx
- Supplemental\_Table\_S10.docx
- Supplemental\_Table\_S11.docx
- Supplemental\_Table\_S12.docx
- Supplemental\_Table\_S13.docx
- Supplemental\_Table\_S14.docx
- Supplemental\_Table\_S3\_JD\_edit.xlsx
- Supplemental\_Table\_S4\_JD\_edit.xlsx
- Supplemental\_Table\_S5\_JD\_edit.xlsx
- Supplemental\_Table\_S6\_JD\_edit.xlsx
