# Supplementary material for: The genomic basis of evolutionary differentiation among honey bees
Source: Genome Res. 2021 Jul;31(7):1203–15. doi: 10.1101/gr.272310.120 (PMC8256857; doi:10.1101/gr.272310.120)
Supplement: Supplemental Material [file supp_gr.272310.120_Supplemental_Fig_S1.pdf]

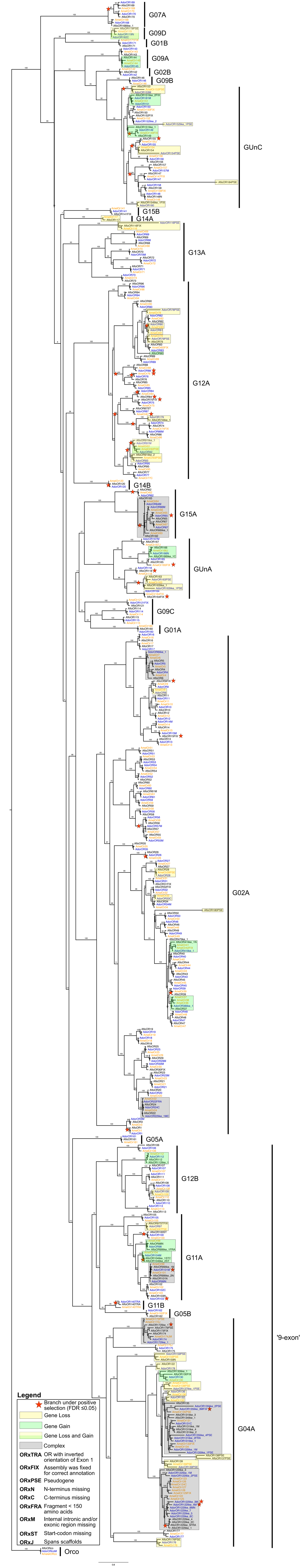

G07A
G09D
G01B
G09A
G02B
G09B
GUnC
G15B
G14A
G13A
G12A
G14B
G15A
GUnA
G09C
G01A
G02A
G05A
G12B
G11A
G11B
G05B
'9-exon'
G04A
Orco
Legend
Branch under positive selection (FDR ≤0.05)
Gene Loss
Gene Gain
Gene Loss and Gain
Complex
ORxTRA OR with inverted orientation of Exon 1
ORxFIX Assembly was fixed for correct annotation
ORxPSE Pseudogene
ORxN N-terminus missing
ORxC C-terminus missing
ORxFRA Fragment < 150 amino acids
ORxM Internal intronic and/or exonic region missing
ORxST Start-codon missing
ORxJ Spans scaffolds

Supplemental Figure S1: Phylogenetic relationships of the honey bee odorant receptors (ORs). The 25 subfamilies (Brand and Ramirez 2017) are labeled throughout the tree. Bootstrap supports are indicated for each branch. Stars indicate branches and ORs under positive selection. Gene losses, gene gains, and more complex evolutionary gene family dynamics are highlighted by yellow, green, and grey boxes, respectively.
