# Supplementary material for: The genomic basis of evolutionary differentiation among honey bees
Source: Genome Res. 2021 Jul;31(7):1203–15. doi: 10.1101/gr.272310.120 (PMC8256857; doi:10.1101/gr.272310.120)
Supplement: Supplemental Material [file supp_gr.272310.120_Supplemental_Fig_S2.pdf]

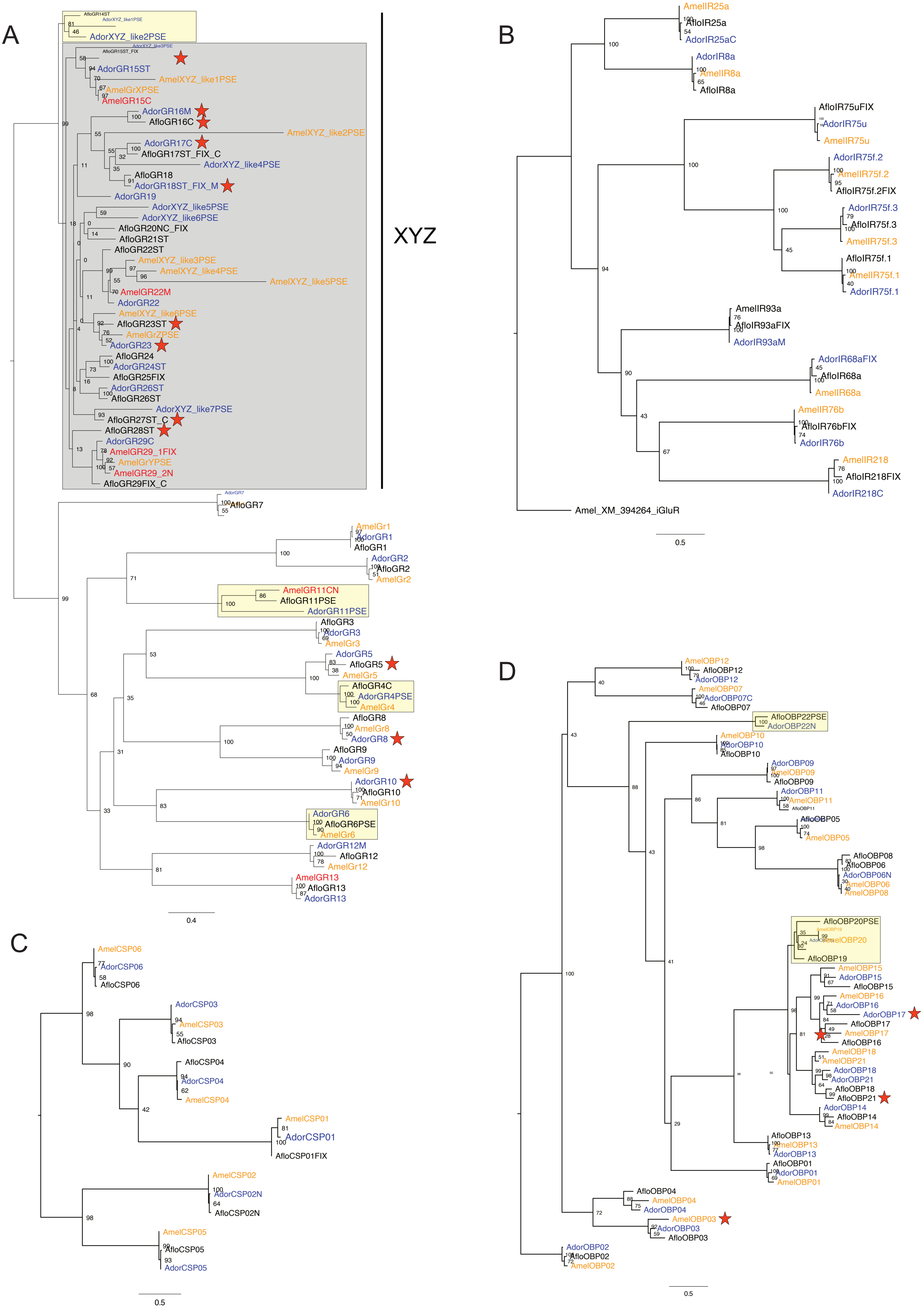

A
AfloGR14ST
AdorXYZ_like1PSE
AdorXYZ_like2PSE
AdorXYZ_like3PSE
AfloGR15ST_FIX
AdorGR15ST
AmelXYZ_like1PSE
AmelGrXPSE
AmelGR15C
AdorGR16M
AfloGR16C
AmelXYZ_like2PSE
AdorGR17C
AfloGR17ST_FIX_C
AdorXYZ_like4PSE
AfloGR18
AdorGR18ST_FIX_M
AdorGR19
AdorXYZ_like5PSE
AdorXYZ_like6PSE
AfloGR20NC_FIX
AfloGR21ST
AfloGR22ST
AmelXYZ_like3PSE
AmelXYZ_like4PSE
AmelXYZ_like5PSE
AmelGR22M
AdorGR22
AmelXYZ_like6PSE
AfloGR23ST
AmelGrZPSE
AdorGR23
AfloGR24
AdorGR24ST
AfloGR25FIX
AdorGR26ST
AfloGR26ST
AdorXYZ_like7PSE
AfloGR27ST_C
AfloGR28ST
AdorGR29C
AmelGR29_1FIX
AmelGrYPSE
AmelGR29_2N
AfloGR29FIX_C
XYZ
AdorGR7
AfloGR7
AmelGr1
AdorGR1
AfloGR1
AdorGR2
AfloGR2
AmelGr2
AmelGR11CN
AfloGR11PSE
AdorGR11PSE
AfloGR3
AdorGR3
AmelGr3
AdorGR5
AfloGR5
AmelGr5
AfloGR4C
AdorGR4PSE
AmelGr4
AfloGR8
AmelGr8
AdorGR8
AfloGR9
AdorGR9
AmelGr9
AdorGR10
AfloGR10
AmelGr10
AdorGR6
AfloGR6PSE
AmelGr6
AdorGR12M
AfloGR12
AmelGr12
AmelGR13
AfloGR13
AdorGR13
0.4
B
AmelIR25a
AfloIR25a
AdorIR25aC
AdorIR8a
AmelIR8a
AfloIR8a
AfloIR75uFIX
AdorIR75u
AmelIR75u
AdorIR75f.2
AmelIR75f.2
AfloIR75f.2FIX
AdorIR75f.3
AfloIR75f.3
AmelIR75f.3
AfloIR75f.1
AmelIR75f.1
AdorIR75f.1
AmelIR93a
AfloIR93aFIX
AdorIR93aM
AdorIR68aFIX
AfloIR68a
AmelIR68a
AmelIR76b
AfloIR76bFIX
AdorIR76b
AmelIR218
AfloIR218FIX
AdorIR218C
Amel_XM_394264_iGluR
0.5
C
AmelCSP06
AdorCSP06
AfloCSP06
AdorCSP03
AmelCSP03
AfloCSP03
AfloCSP04
AdorCSP04
AmelCSP04
AmelCSP01
AdorCSP01
AfloCSP01FIX
AmelCSP02
AdorCSP02N
AfloCSP02N
AmelCSP05
AfloCSP05
AdorCSP05
0.5
D
AmelOBP12
AfloOBP12
AdorOBP12
AmelOBP07
AdorOBP07C
AfloOBP07
AfloOBP22PSE
AdorOBP22N
AmelOBP10
AdorOBP10
AfloOBP10
AdorOBP09
AmelOBP09
AfloOBP09
AdorOBP11
AmelOBP11
AfloOBP11
AfloOBP05
AmelOBP05
AfloOBP08
AfloOBP06
AdorOBP06N
AmelOBP06
AmelOBP08
AfloOBP20PSE
AmelOBP19
AmelOBP20
AfloOBP19
AmelOBP15
AdorOBP15
AfloOBP15
AmelOBP16
AdorOBP16
AdorOBP17
AfloOBP17
AmelOBP17
AfloOBP16
AmelOBP18
AmelOBP21
AdorOBP18
AdorOBP21
AfloOBP18
AfloOBP21
AdorOBP14
AfloOBP14
AmelOBP14
AfloOBP13
AmelOBP13
AdorOBP13
AfloOBP01
AdorOBP01
AmelOBP01
AfloOBP04
AmelOBP04
AdorOBP04
AmelOBP03
AdorOBP03
AfloOBP03
AdorOBP02
AfloOBP02
AmelOBP02
0.5

Supplemental Figure S2: Phylogenetic relationships of the four non-OR chemosensory gene families. A) Gustatory receptors (GRs) excluding ca. 50 small fragments with homology to GRs present in all honey bee species (main text). B) Ionotropic receptors (IRs), C) Chemosensory proteins (CSPs). D) Odorant binding proteins (OBPs). Bootstrap supports are indicated for all branches. Stars indicate branches and ORs under positive selection. Gene losses, gene gains, and more complex evolutionary gene family dynamics are highlighted by yellow, green, and grey boxes, respectively.
