# Supplementary material for: The genomic basis of evolutionary differentiation among honey bees
Source: Genome Res. 2021 Jul;31(7):1203–15. doi: 10.1101/gr.272310.120 (PMC8256857; doi:10.1101/gr.272310.120)
Supplement: Supplemental Material [file supp_gr.272310.120_Supplemental_Methods.docx]

**Material and Methods**

Genome Annotation

**Repeat Detection and Repeatmasking**

We used RepeatModeler 1.0.5 for *de novo* detection of repeats in the three genome assemblies (Ador_1.3, Aflo_1.0, Amel_4.5). For each species, we created a custom repeat library for repeatmasking by combining consensus sequences of known repeat families identified using RepeatModeler with arthropod repeats from the RepBase RepeatMasker library (20130422). Unknown repeat families identified with RepeatModeler were excluded from the repeatmasking library because copy numbers were low and some of these sequences were from paralogous genes. We used RepeatMasker with the custom libraries to repeatmask the assemblies with softmasking. We used the -nolow option to avoid masking low-complexity regions, since some low GC-content regions of Apis genomes may be recognized as low complexity even though they contain genes.

**Generating RNA-seq alignments, transcript assemblies and intron hints**

*A. mellifera* RNA-seq alignments were used to create intron hints files for Augustus and to create transcript assemblies to use in the EVM and MAKER pipelines. We downloaded 107 paired-end and 50 single-end *Apis mellifera* RNA-seq runs with a minimum 100nt read length from the NCBI Sequence Read Archive. We used FastQC (Bioinformatics)⁠ to check data quality and identify adaptors; subsequently we eliminated 17 paired-end and 10 single-end runs. Fastq-mcf (Aronesty 2011)⁠ and Dynamictrim (Cox et al. 2010)⁠ were used to trim adaptors and poor quality sequences, respectively. Tophat2 (Kim et al. 2013)⁠ was used to align reads to the Amel_4.5 genome assembly using default parameters, except: --min-intron-length 30 --microexon-search --min-segment-intron 30 --min-coverage-intron 30. Intron hints were then generated following the methods provided on the Augustus website (<http://augustus.gobics.de/binaries/readme.rnaseq.html>). Transcript assemblies were generated for each RNA-seq alignment dataset using StringTie (Pertea et al. 2015)⁠.

**Annotating the *A. dorsata* and *A. florea* genomes with EVM**

Protein alignments for input to EVM

UniRef90 (Suzek et al. 2015)⁠ and 21,772 *A. mellifera* model RefSeq protein-sequences (<http://ncbi.nlm.nih.gov/genome/annotation_euk/Apis_mellifera/102/>; Jan 2014) were split-mapped to the *A. dorsata* and *A. florea* genomes using the program SPALN2 (Iwata and Gotoh 2012)⁠ with *A. mellifera*-specific parameters. We also used the spliced-protein alignment tool Exonerate protein2genome (Slater and Birney 2005)⁠ to map the *A. mellifera* protein sequences to the *A. dorsata* and *A. florea* genomes. These protein alignments were later used in the EVM pipeline, described below.

Transcript alignment and assembly for Training Sets and input to EVM

Transcript evidence used in the EVM pipelines for both *A. dorsata* and *A. florea* was built from an initial set of 763,591 sequences from several different sources: 1) all *A. mellifera*, *A. florea* and *A. dorsata* mRNA transcripts (GNOMON annotations deemed to be protein-coding) found in the NCBI (GNOMON annotations deemed to be protein-coding) found in the NCBI (<ftp://ftp.ncbi.nlm.nih.gov/genomes/Apis_mellifera/RNA/>, <ftp://ftp.ncbi.nlm.nih.gov/genomes/Apis_florea/RNA> and <ftp://ftp.ncbi.nlm.nih.gov/genomes/Apis_dorsata/RNA/>); 2) the seven-tissue Newbler derived *A. mellifera* transcriptomes that had been used to generate *A. mellifera* OGSv3.2 (Elsik et al. 2014)⁠ 3) the set of *A. mellifera* StringTie transcripts described above. We used the PASA pipeline (r2014-04-17) (Haas et al. 2003)⁠ to assemble transcripts for each species, resulting in 52,618 PASA transcript assemblies for *A. dorsata* and 40,470 PASA transcript assemblies for *A. florea*. Subsequently, PASA was used to run GMAP and BLAT to map the transcriptomes to the respective assemblies and discard transcripts that aligned with less that 95% identity or less than 95% alignment coverage. This filtering step resulted in 32,882 and 27,280 PASA-generated potential training-set gene models longer than 450bp for *A. dorsata* and *A. florea*, respectively.

Training Sets

The initial training sets for *A. dorsata* and *A. florea* were generated using one of the modules of the PASA pipeline. These training-set genes were selected if they aligned over the full-length of annotated protein sequences of the NCBI non-redundant (NR) database (using the algorithm BLASTP, E=10-3, minimum identity=25%; NR database version of Feb. 2014). The *A. dorsata* training set comprised 2,491 gene models randomly selected from within the set of 13,941 longest ORFs (more than 150 amino-acids) corresponding to complete/non-overlapping genes produced by PASA, and the *A. florea* training set comprised 2,481 gene models randomly selected from within the set of 18,737 longest ORFs selected with the same criteria. Of these gene models 80% (1,992 and 1,984 for *A. dorsata* and *A. florea*, respectively) were used to train most of the ab initio tools used as input for EVM in this study, while the remaining 20% (499 and 497 for *A. dorsata* and *A. florea*, respectively) were set aside to test the accuracy of the newly developed matrices. The 2,491 *A. dorsata* training-set protein-coding gene models included 11,851 canonical donor splice sites, 11,906 canonical acceptor sites and 1,981 start codons, and the 2,481 *A. florea* training-set protein-coding gene models included 10,709 canonical donor and 10,817 canonical acceptor sites and 1,975 start codons.

Obtaining geneid gene predictions for input to EVM

Geneid (Alioto et al. 2018)⁠ is an ab initio gene prediction program used to find potential protein-coding genes in anonymous genomic sequences. In the context of geneid training basically consists of computing position weight matrices (PWMs) or Markov models of order 1 for splice sites and start codons, and deriving a model of coding DNA (generally a Markov model of order 5). Furthermore, once a preliminary species-specific matrix is obtained it is further optimized by adjusting two internal matrix parameters: the cutoff of the scores of the predicted exons (eWF) and the ratio of signal to coding statistics information to be used (oWF).

The start codons identified in creation of the training sets (described above) were used to compute position weight matrices (PWMs) while the donor and acceptors were employed to derive Markov matrices of order 1. We also had enough coding/non-coding nucleotides to derive a Markov of order 5 as a model for the coding potential. Accuracy of the geneid parameter files were tested on an evaluation “artificial scaffold”, consisting of the evaluation-set (described above) concatenated gene models with 800 nucleotides of intervening sequence between each of the genes (Supplemental Tables S7, S8).

Geneid can also use external information, such as the coordinates of known introns, to improve the accuracy of its predictions. In order to take advantage of this feature of geneid we extracted all introns corresponding to the protein-coding sequence from the 32,882 and 27,280 PASA-generated potential training-set gene models for *A. dorsata* and *A. florea*, respectively. This resulted in a set of 55,582 introns for *A. dorsata* and 51,929 introns for *A. florea*. To measure the accuracy of the geneid gene predictions when using intronic evidence we mapped all available transcript evidence to the artificial scaffold using the PASA pipeline, used the program’s “training set”-building module to mimic creating a set of PASA transcripts mapping to the test gene models and extracted the introns corresponding the protein-coding sequences. Subsequently, we calculated the accuracy of geneid (+introns) on the test scaffold, which showed an improvement in the performance of the program when using introns as evidence (Supplemental Tables S7, S8).

The training of geneid to obtain a parameter files for *A. dorsata* and *A. florea* was based on the method described to obtain a Drosophila melanogaster geneid parameter file (Parra et al. 2000)⁠. Training was performed in a “semi-automated“ fashion by employing an in–house geneid training tool (geneidTRAINer1.1).

Obtaining SGP2 gene predictions for input to EVM

SGP2 (Parra et al. 2003)⁠ is a syntenic gene prediction tool that combines ab initio gene prediction (geneid) with TBLASTX searches between two or more genome sequences to provide both sensitive and specific gene predictions, and it tends to improve geneid’s performance, especially by reducing the number of false-positive predictions. SGP2 requires a reference genome to which the target genome (in this case *A. dorsata* of *A. florea*) is compared. We decided to use the genomes of three different wasp (*Nasonia vitripennis*, *Nasonia longicornis* and *Nasonia giraulti*) as reference to develop both an SGP2 parameter file for this species. *Nasonia vitripennis*, *Nasonia longicornis* and *Nasonia giraulti* were selected not only because of the relatively good quality of their assemblies but also due to their being at an “appropriate” evolutionary distance from the genome being annotated. This evolutionary distance results in the coding regions of the genes having higher conservation than the intergenic and intronic regions of the target/reference genomes being aligned. This feature contributes to the higher accuracy of SGP2 when compared to geneid, especially the level of specificity.

The starting points for obtaining parameter files for SGP2 were the previously described geneid matrices. The geneid-derived SGP2 parameter files were optimized on “artificial scaffolds” comprising the same set of sequences used to train geneid, concatenated so that 800 “intergenic” nucleotides flanked each of the gene models. SGP2 matrices were optimized by modifying, not only the eWF internal parameter (as done for geneid), but also two SGP2-specific internal parameters (“NO_SCORE” and “HSP_factor”). The “NO_SCORE” parameter provides a penalty for no overlap between TBLASTX-derived HSPs (High-Scoring Pairs) and geneid ab initio predictions in the same region whereas the “HSP_factor” parameter reduces the score assigned to the HSPs in order to maximize the prediction accuracy. The optimal values of “NO_SCORE” and “HSP_factor” were found to be 0.07 and -0.075 respectively for both *A. dorsata* and *A. florea*. The newly developed SGP2 parameter file was evaluated on the same “artificial scaffold” used to test the geneid matrix (Supplemental Tables S7, S8).

SGP2 can also use external information (i.e. introns) improve the accuracy of its predictions. We used the same set of introns as external evidence for SGP2 as we had for geneid. Again the accuracy of SGP2 (+introns) on the test scaffold confirmed an improvement in the performance of the program when introns are used as evidence (Supplemental Tables S7, S8).

The strategy used to obtain the SGP2-specific parameter file was based on the methodology described by Parra et al. (Parra et al. 2003)⁠ used to obtain a human SGP2 parameter file using mouse homology evidence.

Geneid/SGP2 (both with or without introns), augustus (with or without “hints”), glimmerHMM, geneMark and SNAP, using their newly developed *A. florea*-specific parameter files (except for SNAP which used an *A. mellifera* matrix) were subsequently used to predict genes on the latest repeat-masked assembly of this genome (Aflo_1.0_assembly_known_only_nolow.masked.fa).

Obtaining glimmerHMM gene predictions for input to EVM

We also obtained *A. dorsata*-specific and *A. florea*-specific matrices for the gene prediction tool glimmerHMM (Majoros et al. 2004)⁠. GlimmerHMM is an ab initio program that is based on a Generalized Hidden Markov Model (GHMM). This program also incorporates splice site models adapted from the GeneSplicer program and a decision tree adapted from glimmmerM.

We generated the species-specific matrices for this program by using a “self-training” script obtained from the University of Maryland (<http://www.cbcb.umd.edu/software/GlimmerHMM>). The training was performed by following the instructions provided in <http://www.cbcb.umd.edu/software/GlimmerHMM/man.shtml#training>. GlimmerHMM was trained on the same set of sequences used to train the gene prediction programs geneid and SGP2. The new glimmerHMM matrices were evaluated on the same “artificial scaffold” used to evaluate the other ab initio gene prediction parameter files described in this text (Supplemental Tables S7, S8).

Obtaining geneMark gene predictions for input to EVM

We generated *A. dorsata*-specific and *A. florea*-specific matrices for the gene prediction program geneMark (Lomsadze et al. 2005)⁠. The GeneMark-ES algorithm was developed for finding protein-coding genes in eukaryotic genomes without training sets. GeneMark-ES determines species-specific gene finding parameters using a self-training algorithm based on the species of interest genomic sequence. We generated an species-specific matrices for this program by using 15 MBases of *A. dorsata* or *A. florea* genomic sequence and following the self-training instructions found both at the program developer’s web site (<http://opal.biology.gatech.edu/>) and as indicated by Lomsadze et al. (Lomsadze et al. 2005)⁠. GeneMark was trained on the same set of sequences used to train the gene prediction programs described above. The new geneMark matrices were also evaluated on the same artificial scaffolds used to evaluate the other ab initio gene prediction parameter files used in this study (Supplemental Tables S7, S8).

Obtaining Augustus gene predictions for input to EVM

We also built an *A. dorsata*-specific and *A. florea*-specific parameter files for the gene prediction program Augustus (Stanke et al. 2006)⁠. Augustus is a program that predicts genes in eukaryotic genomic sequences and that is also “re-trainable”. The program is based on a Hidden Markov Model and integrates a number of known methods and sub-models. In order to obtain parameter files for Augustus we employed its own training program ([http://www.molecularevolution.org/molevolfiles/exercises/augustus/training.html](http://ncbi.nlm.nih.gov/genome/annotation_euk/Apis_mellifera/102/)), and used it to estimate the optimal parameters for *A. dorsata* given the same 1,992 species-specific genes used in training the other ab initio tools used to obtain gene predictions on these species. The resulting augustus parameter file was evaluated on the same “artificial scaffold” consisting of the 499 concatenated gene models with 800 nucleotides of intervening sequence used to evaluate the other programs previously described (Supplemental Tables S7, S8).

We took advantage of Augustus’ potential to use external evidence to improve its performance. We did this by obtaining a set of predictions that used the newly developed Augustus parameters file in combination with PASA-derived transcript evidence (52,618 and 40,470 PASA transcripts for *A. dorsata* and *A. florea*, respectively, described above). Our strategy for taking advantage of the large set of transcripts followed the methodology described in (<http://augustus.gobics.de/binaries/readme.rnaseq.html>) and in an article by Stanke et al. (21) and allowed us to obtain a higher-accuracy evidence-based set of Augustus(+hints) predictions on the *A. dorsata* and *A. florea* assemblies (Supplemental Tables S7, S8). In order for the Augustus matrices to take advantage of the external data we first had to optimize some internal parameters of the new Augustus parameter file; the exonpart bonus for hints corresponding to PASA-evidence (“E”) was given a bonus of 1×E3. Also, for every exon part that was not supported by the PASA evidence, the probability of the gene structure was given a “malus” or a penalization of 0.997. Furthermore, complete exons predicted by Augustus that perfectly matched the exons in the external hints were given a bonus of 1×E4. The intron bonus for (PASA) hints of source E was set to 1×E5, meaning that a predicted intron would get this bonus when being exactly as in the PASA “hint”.

Obtaining SNAP gene predictions for input to EVM

Our final source of ab initio gene predictions to be used by the EVM combiner was obtained using the program SNAP (Korf 2004)⁠ with its pre-existing *A. mellifera*-specific matrix.

EVM-based annotation by combining different sources

Evidence Modeler (EVM r2012-06-25) (Haas et al. 2008)⁠ was used to obtain consensus coding sequence (CDS) models using three main sources of evidence: aligned transcripts, aligned proteins, and gene predictions. Transcript and protein alignments described above were filtered as suggested in the EVM documentation (<http://evidencemodeler.sourceforge.net/>). Gene predictions were modified as recommended (<http://evidencemodeler.sourceforge.net/>)) and added to the EVM pipeline. Subsequently the transcript alignments, protein alignments and the ab initio gene models were combined into consensus CDS models by EVM using different weights. These weights (Supplemental Tables S9, S10) were selected following the EVM documentation (i.e. <http://evidencemodeler.sourceforge.net/>) and, with regard to the ab initio predictions, also based on the accuracy of the different programs in predicting sequences in the evaluation “artificial scaffold” for this species (Supplemental Tables S7, S8).

The EVM pipeline resulted in 12,176 and 14,420 consensus gene models for *A. dorsata* and *A. florea*, respectively. Sources of evidence (ab initio, protein or transcript) EVM used to build the consensus gene models are provided in Supplemental Tables S11, S12. Final EVM sets after removing gene models with in-frame stop codons consisted of 12,172 and 14,393 gene models for *A. dorsata* and *A. florea*, respectively. The final sets were then updated with UTRs and alternative exons through four rounds of PASA’s routine to update annotations. The resulting transcripts were grouped into genes and then a pre-selected species-specific identifier was assigned to the genes, transcripts and protein products derived from them.

EVM consensus annotation statistics

Finally, and as a quality control, the protein products obtained from the final EVM gene stets were aligned against either the exhaustive NCBI non-redundant (NR-201402) database using BLASTP (E-value < .001 with a minimum identity of 25%) to determine what percentage of the annotated genes matched publicly available sequences. Results showed that ~90% of *A. dorsata* and ~91.5% of *A. florea* consensus EVM protein sequences matched an NR protein given the criteria above. Supplemental tables S13 and S14 provide additional statistics of the EVM consensus protein-coding gene sets.

**Gene prediction in three species with AUGUSTUS-CGP**

The softmasked genome sequences of *Apis dorsata*, *Apis florea*, *Apis mellifera*, *Bombus impatiens* and *Bombus terrestris* were aligned using Cactus (Paten et al. 2011)⁠ in two iterations. First, a phylogenetic tree was estimated using ffpry (Sims and Kim 2011)⁠. The resulting tree was manually manipulated to convert negative branch lengths to positive numbers. Cactus was initially run with this first tree. Subsequently, a second phylogenetic tree was computed with Mr. Bayes (Huelsenbeck and Ronquist 2001)⁠. The second tree was used to create a final genome alignment with Cactus.

The gene prediction software AUGUSTUS (Stanke et al. 2008)⁠ was recently extended to predict consistent gene structures in multiple genomes, simultaneously (König et al. 2016)⁠. This novel "AUGUSTUS-CGP" version was applied to the genome alignment using the honeybee1 parameters set (Elsik et al. 2014)⁠ and intron hints from RNA-seq data of *Apis mellifera*. The command line was:

augustus --/CompPred/omegaEff=1 --/CompPred/conservation=1 --treefile=tree.nwk --alnfile=genome.maf --softmasking=on --species=honeybee1 --dbaccess=bees.db --speciesfilenames=genomes.tbl --extrinsicCfgFile=extrinsic.cfg --optCfgFile=cgp_parameters.cfg --/CompPred/ec_addend=-5 --/CompPred/ec_factor=2 --/CompPred/featureScoreHects=1 --/CompPred/outdir=pred --dbhints=true

where input files as follows:

1. The file extrinsic.cfg contained the non-neutral general parameters

(nonexonpart        1 1 M 1 1e+100  RM 1 1.15 E 1 1) and the *A. mellifera* specific non-neutral parameters (intron        1 .34 M 1 1e+100 RM 1 1 E 1) 1e5.

2. The file cgp_parameters.cfg contained the following:

temperature             3

/MeaPrediction/x0_E     -1.25

/MeaPrediction/x0_I     -0.78125

/MeaPrediction/x1_E     50

/MeaPrediction/x1_I     100

/MeaPrediction/y0_E     0.5

/MeaPrediction/y0_I     0.9

/MeaPrediction/alpha_E  5.375

/MeaPrediction/alpha_I  2.5075

/MeaPrediction/i1_E     0

/MeaPrediction/i1_I     0

/MeaPrediction/i2_E     0.99

/MeaPrediction/i2_I     0.99

/MeaPrediction/j1_E     -1.25

/MeaPrediction/j1_I     -0.78125

/MeaPrediction/j2_E     4.9

/MeaPrediction/j2_I     9

/MeaPrediction/r_be     0.5

/MeaPrediction/r_bi     0.5

/CompPred/exon_gain     0.000001

/CompPred/exon_loss     0.000001

/CompPred/dd_factor     20

/CompPred/maxIterations 300

/CompPred/phylo_factor  10

Since predictions at the borders of alignment blocks can be problematic, such genes were joined with single species predictions. For *A. mellifera*, single species predictions were created with AUGUSTUS using the following command:

augustus --species=honeybee1 --hintsfile=intron.hints --extrinsicCfgFile=extrinsic.cfg --softmasking=on genome.fa

where intron.hints contained RNA-seq intron hints (described above) and extrinsic.cfg had the same non-neutral lines as shown for AUGUSTUS-CGP.

Single gene predictions for *B. impatiens* and *B. terrestris* were created using AUGUSTUS with the following command:

augustus --species=bombus_terrestris2 --extrinsicCfgFile=extrinsic.cfg --softmasking=on genome.fa

where bombus_terrestris2 is the parameter set from (Sadd et al. 2015)⁠.

AUGUSTUS-CGP predictions of *A. dorsata* and *A. florea* were joined with EVM gene models, described above.

**Annotating the *A. dorsata* genome with MAKER**

We used the MAKER genome annotation pipeline to choose among the *A. dorsata* gene models annotated by EVM and Augustus-CGP. This application took advantage of MAKER’s annotation management capabilities, rather than its use as a de novo annotation pipeline. The annotations created by EVM and Augustus-CGP were both supplied to MAKER as predictions which would be evaluated by evidential support. The softmasked version of the *A. dorsata* genome, described above, was provided to MAKER. The protein evidence used for evaluating models was the UniRef90 database and the RefSeq *Apis mellifera* proteins, which were aligned to the genome with BLASTX. The transcript evidence used for evaluating the gene models was the StringTie dataset described above. The StringTie gtf files were converted to fasta files with the cufflinks_gtf_genome_to_cdna_fasta.pl script from the Transdecoder package. Because the RNA-seq evidence originated from a different species than was being annotated, these datasets were aligned with TBLASTX instead of BLASTN (ncbi-blast+ v.2.2.30). Annotations that overlap the same region of the genome were evaluated by MAKER based on their support by transcript and protein sequence alignments. At each locus, the annotation that was best supported by evidence was included in the final MAKER set as the model for that region.

**Gene set annotation**

We used InterProScan (Zdobnov and Apweiler 2001)⁠ to compare protein sequences to the following InterPro (Finn et al. 2017)⁠ protein domain and motif databases: Pfam (Finn et al. 2016)⁠, PANTHER (Mi et al. 2017)⁠, TIGRFAMS (Haft et al. 2013)⁠, SMART (Haft et al. 2013)⁠, PRODOM (Haft et al. 2013)⁠, PROSITE (Sigrist et al. 2009)⁠, PIRSF (Wu 2004)⁠, PRINTS (Attwood et al. 2003)⁠, SUPERFAMILY (Oates et al. 2015).⁠

**Bacterial Contamination**

InterPro results for some of the genes specific to only one or two *Apis* species suggested that they may be a result of bacterial contamination in an *Apis* genome assembly. To screen for potential bacterial contamination in the three *Apis* genome assemblies, each genome assembly was aligned to the NCBI RefSeq Prokaryotic Representative Genomes database using Megablast (Zhang et al. 2000)⁠ with an E-value threshold of 1 x 10-6. Locations of Megablast hits in the Apis genome assemblies were converted to gff3 format. IntersectBed (BedTools (Quinlan and Hall 2010)⁠) was used to identify gene coordinates that overlapped bacterial alignment coordinates to flag genes that were potentially a result of bacterial sequence contamination in the genome assembly.

**Ortholog prediction**

Protein sequences from the three comparative gene sets were combined into one file that was used in an all by all protein comparison with FASTA (Pearson and Lipman 1988)⁠ using an E-value threshold 0.001. Ortholog families containing one gene from each species were identified using the FASTA output as follows. For each protein query, the identifier of the aligned protein (“hit”) with the highest Smith-Waterman score of each species was mapped to its gene id and saved. At the end of this step, each protein query was associated with up to three best-hit genes: the gene encoding the query itself and a gene from each of the two other species, if present. We defined families of single-copy orthologs as groups containing one gene per species such that every pair of genes in the family had a reciprocal best-hit gene using the Smith-Waterman score for protein alignments. Since some genes code for more than one protein isoform, each isoform of a particular gene was required to be associated with same set of best-hit genes.

After creating the families of *Apis* orthologs, a *Bombus* protein to serve as an outgroup was identified for each family as follows. FASTA was used to compare proteins from the *Apis* ortholog families with proteins of *B. impatiens* (RefSeq and OGS (Sadd et al. 2015)⁠) and *B. terrestris* (RefSeq), using an E-value threshold of 0.001. One best hit *Bombus* protein was selected as an outgroup for each ortholog family, with preference in the following order: *B. impatiens* RefSeq, *B. impatiens* OGS, *B. terrestris* RefSeq. 9310 *Apis* ortholog families were assigned a *Bombus* protein to be used as an outgroup.

**Multiple sequence alignment**

For each ortholog family, the longest protein isoforms for each of the three *Apis* genes and one *Bombus* gene were used in multiple sequence alignment with PRANK (v.150803) (Löytynoja and Goldman 2008)⁠ and unreliably aligned residues were masked with GUIDANCE (v2.02) (Penn et al. 2010)⁠. This combination was shown to perform the best on simulated data (Jordan and Goldman 2012; Privman et al. 2012)⁠. Only gene families including strictly one ortholog in each of the 4 species were selected (8850 gene families). Protein sequences were replaced with coding sequences in the multiple alignments but failed for 109 ortholog families, as coding sequences did not match the predicted proteins. Alignments regions, where gapped positions were present, were removed with Gblocks (v0.91b; type = codons; minimum length of a block = 4; no gaps allowed) (Talavera and Castresana 2007)⁠, as these are the most problematic for positive selection inference (Fletcher and Yang 2010; Markova-Raina and Petrov 2011)⁠. Finally, CDS shorter than 303 nucleotides (101 codons) were eliminated, we used here a more stringent filter than in Hambuch & Parsch (Hambuch and Parsch 2005)⁠ as our phylogenetic depth is limited.

**Tests of functional category enrichment**

To account for possible biases in GO annotations resulting from our alignment filtering, we performed a Fisher’s exact test between the full and filtered data-sets and found the following depleted GO annotations: “dendrite membrane” (CC), P < 0.0001; “odorant binding” (MF), P < 0.0001; “olfactory receptor activity” (MF), P < 0.0001; “detection of chemical stimulus involved in sensory perception of smell” (BP), P < 0.0001; “mitochondrial cytochrome c oxidase assembly” (BP), P < 0.1; “mitochondrial intermembrane space” (CC), P < 0.2; “outer dynein arm” (CC), P < 0.2 and the following enriched GO annotations: “protein binding” (MF), P < 0.01; “cytosol” (CC), P < 0.02; “Golgi membrane” (CC), P < 0.2). Thus, we disregarded our finding that the GO term “cytosol” was over-enriched as a potential artifact.

To identify functional categories enriched with genes under positive selection, the SUMSTAT test based on the test from Efron and Tibshirani (2007) (Supplemental Code) was used as described in Roux et al. (2014). The SUMSTAT test is more sensitive than other methods, and minimizes the rate of false positives (Ackermann and Strimmer 2009; Tintle et al. 2009; Fehringer et al. 2012; Daub et al. 2013)⁠. To be able to use the distribution of log-likelihood ratios of the positive selection test as scores in the SUMSTAT test, a fourth root transformation was used (Roux et al. 2014)⁠. This transformation conserves the ranks of gene families (Canal 2005)⁠.

The list of significant gene sets resulting from enrichment tests is usually highly redundant. We therefore implemented the “elim” algorithm from the Bioconductor package topGO, to decorrelate the graph structure of the Gene Ontology (Alexa et al. 2006)⁠. To account for multiple testing, the final list of p-values resulting from this test was corrected with the FDR (Benjamini and Hochberg 1995; Roux et al. 2014) and set up our significant threshold at 20% as in Roux et al. (2014).

**References**

Ackermann M, Strimmer K. 2009. A general modular framework for gene set enrichment analysis. *BMC Bioinformatics* **10**: 47.

Alioto T, Blanco E, Parra G, Guigó R. 2018. Using geneid to Identify Genes. *Curr Protoc Bioinforma* **64**: 1–32.

Aronesty E. 2011. ea-utils : “Command-line tools for processing biological sequencing data.” https://github.com/ExpressionAnalysis/ea-utils.

Attwood TK, Bradley P, Flower DR, Gaulton A, Maudling N, Mitchell AL, Moulton G, Nordle A, Paine K, Taylor P, et al. 2003. PRINTS and its automatic supplement, prePRINTS. *Nucleic Acids Res* **31**: 400–402.

Bioinformatics B. FastQC A quality control tool for high throughput sequence data. https://www.bioinformatics.babraham.ac.uk/projects/fastqc/.

Canal L. 2005. A normal approximation for the chi-square distribution. *Comput Stat Data Anal* **48**: 803–808.

Cox MP, Peterson DA, Biggs PJ. 2010. SolexaQA: At-a-glance quality assessment of Illumina second-generation sequencing data. *BMC Bioinformatics* **11**.

Daub JT, Hofer T, Cutivet E, Dupanloup I, Quintana-Murci L, Robinson-Rechavi M, Excoffier L. 2013. Evidence for polygenic adaptation to pathogens in the human genome. *Mol Biol Evol* **30**: 1544–1558.

Efron B and Tibshirani R. 2007. On testing the significance of sets of genes. *Ann Appl Stat* **1**:107–129.

Elsik CG, Worley KC, Bennett AK, Beye M, Camara F, Childers CP, Graaf DC De, Debyser G, Deng J, Devreese B, et al. 2014. Finding the missing honey bee genes: lessons learned from a genome upgrade. *BMC Genomics* **15**: 86.

Fehringer G, Liu G, Briollais L, Brennan P, Amos CI, Spitz MR, Bickeböller H, Wichmann HE, Risch A, Hung RJ. 2012. Comparison of pathway analysis approaches using lung cancer GWAS data sets. *PLoS One* **7**: e31816.

Finn RD, Coggill P, Eberhardt RY, Eddy SR, Mistry J, Mitchell AL, Potter SC, Punta M, Qureshi M, Sangrador-Vegas A, et al. 2016. The Pfam protein families database: Towards a more sustainable future. *Nucleic Acids Res* **44**: D279–D285.

Fletcher W, Yang Z. 2010. The effect of insertions, deletions, and alignment errors on the branch-site test of positive selection. *Mol Biol Evol* **27**: 2257–2267.

Haas BJ, Delcher AL, Mount SM, Wortman JR, Smith RK, Hannick LI, Maiti R, Ronning CM, Rusch DB, Town CD, et al. 2003. Improving the Arabidopsis genome annotation using maximal transcript alignment assemblies. *Nucleic Acids Res* **31**: 5654–5666.

Haas BJ, Salzberg SL, Zhu W, Pertea M, Allen JE, Orvis J, White O, Robin CR, Wortman JR. 2008. Automated eukaryotic gene structure annotation using EVidenceModeler and the Program to Assemble Spliced Alignments. *Genome Biol* **9**: 1–22.

Haft DH, Selengut JD, Richter RA, Harkins D, Basu MK, Beck E. 2013. TIGRFAMs and genome properties in 2013. *Nucleic Acids Res* **41**: 387–395.

Hambuch TM, Parsch J. 2005. Patterns of synonymous codon usage in *Drosophila melanogaster* genes with sex-biased expression. *Genetics* **170**: 1691–1700.

Huelsenbeck JP, Ronquist F. 2001. MRBAYES: Bayesian inference of phylogenetic trees. *Bioinformatics* **17**: 754–755.

Iwata H, Gotoh O. 2012. Benchmarking spliced alignment programs including Spaln2, an extended version of Spaln that incorporates additional species-specific features. *Nucleic Acids Res* **40**: 1–9.

Kim D, Pertea G, Trapnell C, Pimentel H, Kelley R, Salzberg SL. 2013. TopHat2: accurate alignment of transcriptomes in the presence of insertions, deletions and gene fusions. *Genome Biol* **14**: R36. http://genomebiology.biomedcentral.com/articles/10.1186/gb-2013-14-4-r36.

König S, Romoth LW, Gerischer L, Stanke M. 2016. Simultaneous gene finding in multiple genomes. *Bioinformatics* **32**: 3388–3395.

Korf I. 2004. Gene finding in novel genomes. *BMC Bioinformatics* **5**: 1–9.

Jordan G, Goldman N. 2012. The effects of alignment error and alignment filtering on the sitewise detection of positive selection. *Mol Biol Evol* **29**: 1125–1139.

Lomsadze A, Ter-Hovhannisyan V, Chernoff YO, Borodovsky M. 2005. Gene identification in novel eukaryotic genomes by self-training algorithm. *Nucleic Acids Res* **33**: 6494–6506.

Majoros WH, Pertea M, Salzberg SL. 2004. TigrScan and GlimmerHMM: Two open source ab initio eukaryotic gene-finders. *Bioinformatics* **20**: 2878–2879.

Markova-Raina P, Petrov D. 2011. High sensitivity to aligner and high rate of false positives in the estimates of positive selection in the 12 *Drosophila* genomes. *Genome Res* **21**: 863–874.

Mi H, Huang X, Muruganujan A, Tang H, Mills C, Kang D, Thomas PD. 2017. PANTHER version 11: Expanded annotation data from gene ontology and reactome pathways, and data analysis tool enhancements. *Nucleic Acids Res* **45**: D183–D189.

Oates ME, Stahlhacke J, Vavoulis D V., Smithers B, Rackham OJL, Sardar AJ, Zaucha J, Thurlby N, Fang H, Gough J. 2015. The SUPERFAMILY 1.75 database in 2014: A doubling of data. *Nucleic Acids Res* **43**: D227–D233.

Parra G, Agarwal P, Abril JF, Wiehe T, Fickett JW, Guigó R. 2003. Comparative gene prediction in human and mouse. *Genome Res* **13**: 108–117.

Parra G, Blanco E, Guigó R. 2000. GeneId in Drosophila. *Genome Res* **10**: 511–515.

Paten B, Earl D, Nguyen N, Diekhans M, Zerbino D, Haussler D. 2011. Cactus: Algorithms for genome multiple sequence alignment. *Genome Res* **21**: 1512–1528.

Pertea M, Pertea GM, Antonescu CM, Chang TC, Mendell JT, Salzberg SL. 2015. StringTie enables improved reconstruction of a transcriptome from RNA-seq reads. *Nat Biotechnol* **33**: 290–295.

Privman E, Penn O, Pupko T. 2012. Improving the performance of positive selection inference by filtering unreliable alignment regions. *Mol Biol Evol* **29**: 1–5.

Quinlan AR, Hall IM. 2010. BEDTools: A flexible suite of utilities for comparing genomic features. *Bioinformatics* **26**: 841–842.

Roux J, Privman E, Moretti S, Daub JT, Robinson-Rechavi M, Keller L. 2014. Patterns of positive selection in seven ant genomes. *Mol Biol Evol* **31**: 1661–1685.

Sadd BM, Barribeau SM, Bloch G, de Graaf DC, Dearden P, Elsik CG, Gadau J, Grimmelikhuijzen CJP, Hasselmann M, Lozier JD, et al. 2015. The genomes of two key bumblebee species with primitive eusocial organization. *Genome Biol* **16**: 76.

Sigrist CJA, Cerutti L, De Castro E, Langendijk-Genevaux PS, Bulliard V, Bairoch A, Hulo N. 2009. PROSITE, a protein domain database for functional characterization and annotation. *Nucleic Acids Res* **38**: 161–166.

Sims GE, Kim SH. 2011. Whole-genome phylogeny of Escherichia coli/Shigella group by feature frequency profiles (FFPs). *Proc Natl Acad Sci U S A* **108**: 8329–8334.

Slater GSC, Birney E. 2005. Automated generation of heuristics for biological sequence comparison. *BMC Bioinformatics* **6**: 1–11.

Stanke M, Diekhans M, Baertsch R, Haussler D. 2008. Using native and syntenically mapped cDNA alignments to improve de novo gene finding. *Bioinformatics* **24**: 637–644.

Stanke M, Keller O, Gunduz I, Hayes A, Waack S, Morgenstern B. 2006. AUGUSTUS: A b initio prediction of alternative transcripts. *Nucleic Acids Res* **34**: 435–439.

Suzek BE, Wang Y, Huang H, McGarvey PB, Wu CH. 2015. UniRef clusters: A comprehensive and scalable alternative for improving sequence similarity searches. *Bioinformatics* **31**: 926–932.

Talavera G, Castresana J. 2007. Improvement of phylogenies after removing divergent and ambiguously aligned blocks from protein sequence alignments. *Syst Biol* **56**: 564–577.

Tintle NL, Borchers B, Brown M, Bekmetjev A. 2009. Comparing gene set analysis methods on single-nucleotide polymorphism data from Genetic Analysis Workshop 16. *BMC Proc* **3**: S96.

Wu CH. 2004. PIRSF: family classification system at the Protein Information Resource. *Nucleic Acids Res* **32**: 112D – 114.

Zhang Z, Schwartz S, Wagner L, Miller W. 2000. A greedy algorithm for aligning DNA sequences. *J Comput Biol* **7**: 203–214.
