# Supplementary material for: The genomic basis of evolutionary differentiation among honey bees
Source: Genome Res. 2021 Jul;31(7):1203–15. doi: 10.1101/gr.272310.120 (PMC8256857; doi:10.1101/gr.272310.120)
Supplement: Supplemental Material [file supp_gr.272310.120_Supplemental_Table_S7.docx]

**Supplemental Table S7:** Accuracy of gene prediction on an *A. dorsata* “artificial scaffold” consisting of 499 concatenated *A. dorsata* test sequences (with approximately 800 nucleotides of sequence between each of the gene models) using the *ab initio* programs geneid, augustus, glimmerHMM and geneMark with *A. dorsata* parameter files (*i.e.* “adorsata”) that were built for each program given the same train-set of 1,992 gene models. The exception is geneMark that was trained on 15Mbases of genomic sequence. An additional *ab initio* program (SNAP) was evaluated given predictions obtained using an *A. mellifera* (“amel”) matrix. The accuracy of SGP2 (homology evidence-based prediction tool that used the genomes of 3 wasps genome as reference) and that of Augustus (using RNASeq and transcript evidence i.e. “augustus+hints”) were also tested for accuracy on the same set of sequences. Geneid (geneid+introns) and SGP2 (SGP2+introns) using introns as external evidence were also evaluated. (SN & SP: sensitivity & specificity at nucleotide level; SNe & SPe: sensitivity & specificity at exon level; SNg & SPg: sensitivity & specificity at gene level).

| **Program/param** | **SN** | **SP** | **SNe** | **SPe** | **SNg** | **SPg** |
| --- | --- | --- | --- | --- | --- | --- |
| Geneid adorsata | 0.96 | 0.95 | 0.81 | 0.82 | 0.43 | 0.40 |
| Geneid+intron adorsata | 0.98 | 0.96 | 0.89 | 0.84 | 0.55 | 0.49 |
| SGP2 adorsata / 3 wasps | 0.97 | 0.95 | 0.84 | 0.83 | 0.43 | 0.40 |
| SGP2+intron adorsata / 3 wasps | 0.98 | 0.96 | 0.89 | 0.84 | 0.55 | 0.50 |
| Augustus+hints adorsata | 0.97 | 0.95 | 0.89 | 0.87 | 0.59 | 0.56 |
| Augustus adorsata | 0.93 | 0.92 | 0.79 | 0.78 | 0.36 | 0.37 |
| GlimmerHMM  adorsata | 0.95 | 0.94 | 0.76 | 0.76 | 0.36 | 0.31 |
| GeneMark adorsata | 0.89 | 0.94 | 0.70 | 0.72 | 0.23 | 0.25 |
| SNAP amel | 0.92 | 0.88 | 0.67 | 0.52 | 0.19 | 0.10 |
