# Supplementary material for: The genomic basis of evolutionary differentiation among honey bees
Source: Genome Res. 2021 Jul;31(7):1203–15. doi: 10.1101/gr.272310.120 (PMC8256857; doi:10.1101/gr.272310.120)
Supplement: Supplemental Material [file supp_gr.272310.120_Supplemental_Table_S9.docx]

**Supplemental Table S9:** Weights used by EVM to create a consensus CDS model for *A. dorsata.* (SPLAN2 uniprot90 spaln2 against Uniprot90 proteins; SPALN2 ENSAmel l: SPALN2 against *A. mellifera* ENSEMBL proteins; Exonerate ENSAmel: exonerate against *A. mellifera* ENSEMBL proteins.

| **Type** | **Source** | **Weight** |
| --- | --- | --- |
| *Ab initio* prediction | Augustus | 1.25 |
| *Ab initio* prediction | AugustusHints | 2.25 |
| *Ab initio* prediction | GlimmerHMM | 1.25 |
| *Ab initio* prediction | GeneMark | 1 |
| *Ab initio* prediction | geneid | 1.5 |
| *Ab initio* prediction | sgp2 | 1.75 |
| *Ab initio* prediction | geneid+introns | 2 |
| *Ab initio* prediction | sgp2+introns | 2.25 |
| *Ab initio* prediction | SNAP | 0.5 |
| Protein | SPALN2 uniprot90 | 5 |
| Protein | SPALN2 ENSAmel | 4 |
| Protein | exonerate ENSAmel | 4 |
| Transcript | PASA | 10 |
