# Supplementary material for: The genomic basis of evolutionary differentiation among honey bees
Source: Genome Res. 2021 Jul;31(7):1203–15. doi: 10.1101/gr.272310.120 (PMC8256857; doi:10.1101/gr.272310.120)
Supplement: Supplemental Material [file supp_gr.272310.120_Supplemental_Table_S11.docx]

**Supplemental Table S11:** Break down of the types of evidence used to build the 12,176 gene-models EVM consensus set (prior to filtering for four in-frame stop gene models).

| **Type of source of evidence** | **Number of consensus gene models supported by the type of source of evidence (% of total number of EVM reference gene models supported)** |
| --- | --- |
| PASA transcript alignments | 9,086 (74.61%) |
| Protein alignments | 9,539 (78.33%) |
| Protein OR PASA alignments | 9,785 (80.36%) |
| Protein AND PASA alignments | 8,840 (72.56%) |
| Protein, PASA and at least one source of *ab initio* predictions | 8,840 (72.56%) |
| Exclusively *ab initio* evidence (geneid, geneidi, sgp2, sgp2i, augustus, augustushints or snap) | 2,391 (19.64%) |
| Only one source of *ab initio* predictions (No protein or transcript evidence) | 1,277 (10.49%) |
| At least two sources of *ab initio* evidence (No protein or transcript evidence) | 1,114 (9.15%) |
| All sources of *ab initio* evidence (No protein or transcript evidence) | 125 (1.03%) |
| just geneid, geneidi/spg2, sgp2i (No protein or transcript evidence) | 680(5.58%) |
| just geneid/geneidi (No protein or transcript evidence) | 149 (1.22%) |
| just sgp2/sgp2i (No protein or transcript evidence) | 57 (0.47%) |
| just augustus/augustus+hints (No protein or transcript evidence) | 515 (4.23%) |
| just SNAP (No protein or transcript evidence) | 55 (0.45%) |
| singleEXON genes (with *ab initio* evidence from more than 1 program and/or protein/PASA evidence | 919 (7.22%) |
