# Supplementary material for: The genomic basis of evolutionary differentiation among honey bees
Source: Genome Res. 2021 Jul;31(7):1203–15. doi: 10.1101/gr.272310.120 (PMC8256857; doi:10.1101/gr.272310.120)
Supplement: Supplemental Material [file supp_gr.272310.120_Supplemental_Table_S12.docx]

**Supplemental Table S12:** Break down of the types of evidence used to build the 14,420 gene-models EVM consensus set (prior to filtering for 37 in-frame stop gene models).

| **Type of source of evidence** | **Number of consensus gene models supported by the type of source of evidence (% of total number of EVM reference gene models supported)** |
| --- | --- |
| PASA transcript alignments | 8,925 (61.89%) |
| Protein alignments | 10,077 (69.87%) |
| Protein OR PASA alignments | 10,229 (70.93%) |
| Protein AND PASA alignments | 8,773 (60.83%) |
| Protein, PASA and at least one source of *ab initio* predictions | 8,773 (60.83%) |
| Exclusively *ab initio* evidence (geneid, geneidi, sgp2, sgp2i, augustus, augustushints or snap) | 4,191 (29.06%) |
| Only one source of *ab initio* predictions (No protein or transcript evidence) | 1,940 (13.45%) |
| At least two sources of *ab initio* evidence (No protein or transcript evidence) | 2,251 (15.61%) |
| All sources of *ab initio* evidence (No protein or transcript evidence) | 236 (1.64%) |
| just geneid, geneidi/spg2, sgp2i (No protein or transcript evidence) | 1,072 (7.43%) |
| just geneid/geneidi (No protein or transcript evidence) | 188 (1.30%) |
| just sgp2/sgp2i (No protein or transcript evidence) | 128 (0.89%) |
| just augustus/augustus+hints (No protein or transcript evidence) | 550 (3.81%) |
| just SNAP (No protein or transcript evidence) | 258 (1.79%) |
| singleEXON genes (with *ab initio* evidence from more than 1 program and/or protein/PASA evidence | 1,610 (11.17%) |
