# Supplementary material for: The genomic basis of evolutionary differentiation among honey bees
Source: Genome Res. 2021 Jul;31(7):1203–15. doi: 10.1101/gr.272310.120 (PMC8256857; doi:10.1101/gr.272310.120)
Supplement: Supplemental Material [file supp_gr.272310.120_Supplemental_Table_S13.docx]

**Supplemental Table S13:** Statistics for the EVM-generated protein-coding annotation reference set for *A. dorsata.*

| Annotation version | *A.dorsata 2b* |
| --- | --- |
| Genome length (Mbases) | 230.34 |
| number of scaffolds | 4,040 |
| Number of protein-coding genes | 12,172 |
| Gene density (genes/Kbase) | 0.053 |
| Number of protein-coding transcripts | 23,774 |
| Transcripts/gene (range) (% genes with more than 1 transcript) | 1.95 (SD 2.26) (1 – 43) (32.32%) |
| Number of transcripts with UTRs | 19,275 |
| Number of proteins | 20,508 |
| Number of complete proteins (%) | 19,893 (97.00%) |
| Number/(%) proteins with similarity to sequences in the NCBI NR database (E=10^-3^; min. identity=25%) | 18,896 (92.2%) |
| Avg. length of proteins (range) | 599.44 aa. (SD 687.53) (45 – 19,194) |
| Avg. length of full-length proteins (range) | 603.95 aa. (SD 653.98) (45– 19,194) |
| Number of partial proteins (not starting with "M") | 373 (1.82%) |
| Avg. length of partial proteins (not starting with "M") | 402.68 aa. (SD 731.48) |
| Number of partial proteins (no terminal STOP codon) | 371 (1.81%) |
| Avg. length of partial proteins (no terminal STOP codon) | 397.46 aa. (SD 1,639.63) |
| Number of partial proteins (not starting with an M -and- no terminal STOP codon) | 129 (0.63%) |
| Avg. length of partial proteins (not starting with an M -and- no terminal STOP codon) | 145.67 aa. (SD 334.54) |
| Number of partial proteins (not starting with an M -or- no terminal STOP codon) | 615 (2.99%) |
| Avg. length of partial proteins (not starting with an M -or- no terminal STOP codon) | 453.44 aa. (SD 1,380.72) |
| Number of protein-coding exons | 175,292 |
| Number of introns | 151,518 |
| Number of UTRs (spliced) | 46,337 |
| Number of single-exon genes | 2,323 |
| Number of multi-exonic transcripts (genes) | 21,451 (9,849) |
| Exons/transcript (range) (excludes single-exon genes) | 8.06 (SD 6.76) (2 – 181) |
| Introns/transcript (range) | 7.06 (SD 6.76) (1 – 180) |
| “spliced” UTRs/transcript (range) | 2.40 (SD 0.85) (1 - 9) |
| Avg. length of introns (range) | 1,012.05 (SD 4,299.43) (21 – 194,373) |
| Avg. length of mono-exonic genes | 533.65 (SD 752.04) |
| Avg. length of exons (excludes mono-exonic genes) | 242.45 (SD 340.80) |
| Avg. length of first exons | 231.30 (SD 385.13) |
| Avg. length of internal exons | 246.12 (SD 328.32) |
| Avg. length of terminal exons | 231.40 (SD 366.40) |
| Avg. length of CDS (range) | 1,815.62 (SD 2,032.35) (135 – 57,582) |
| Avg. length of UTRs (range) | 300.22 (SD 417.41) (1 – 6,801) |
| Avg. length of primary transcripts | 9,822.31 (SD 18,622.70) |
| G+C content exonic (mono-exonic genes) | 39.39% (SD 14.64) |
| G+C content exonic (excludes mono-exonic genes) | 37.01% (SD 8.41) |
| G+C content exonic (first exons) | 37.11% (SD 10.77) |
| G+C content exonic (internal exons) | 38.22% (SD 9.77) |
| G+C content exonic (terminal exons) | 37.03% (SD 10.79) |
| G+C content intronic | 18.30% (SD 9.23) |
| G+C content UTRs | 26.60% (SD 10.16) |
