# Supplementary material for: The genomic basis of evolutionary differentiation among honey bees
Source: Genome Res. 2021 Jul;31(7):1203–15. doi: 10.1101/gr.272310.120 (PMC8256857; doi:10.1101/gr.272310.120)
Supplement: Supplemental Material [file supp_gr.272310.120_Supplemental_Table_S14.docx]

**Supplemental Table S14:** Statistics for the EVM-generated protein-coding annotation reference set for *A. florea.*

| Annotation versions | *A.florea 1a* |
| --- | --- |
| Genome length (Mbases) | 230.47 |
| number of scaffolds | 7,945 |
| Number of protein-coding genes | 14,393 |
| Gene density (genes/Kbase) | 0.062 |
| Number of protein-coding transcripts | 21,091 |
| Transcripts/gene (range) (% genes with more than 1 transcript) | 1.47 (SD 1.46) (1 – 23) (18.3%) |
| Number of transcripts with UTRs | 13,226 |
| Number of proteins | 19,173 |
| Number of complete proteins (%) | 17,613 (91.86%) |
| Number/(%) proteins with similarity to sequences in the NCBI NR database (E=10^-3^; min. identity=25%) | 17,551 (91.54%) |
| Avg. length of proteins (range) | 493.11 aa. (SD 553.67) (50 – 17,528) |
| Avg. length of full-length proteins (range) | 518.39 aa. (SD 548.43) (50– 17,528) |
| Number of partial proteins (not starting with "M") | 920 (4.80%) |
| Avg. length of partial proteins (not starting with "M") | 185.74 aa. (SD 181.78) |
| Number of partial proteins (no terminal STOP codon) | 977 (5.1%) |
| Avg. length of partial proteins (no terminal STOP codon) | 211.15 aa. (SD 652.17) |
| Number of partial proteins (not starting with an M -and- no terminal STOP codon) | 337 (1.76%) |
| Avg. length of partial proteins (not starting with an M -and- no terminal STOP codon) | 157.96 aa. (SD 109.21) |
| Number of partial proteins (not starting with an M -or- no terminal STOP codon) | 1,560 (8.14%) |
| Avg. length of partial proteins (not starting with an M -or- no terminal STOP codon) | 207.65 aa. (SD 532.02) |
| Number of protein-coding exons | 129,084 |
| Number of introns | 107,993 |
| Number of UTRs (spliced) | 31,183 |
| Number of single-exon genes | 3,565 |
| Number of multi-exonic transcripts (genes) | 17,526 (10,828) |
| Exons/transcript (range) (excludes single-exon genes) | 7.16 (SD 5.93) (2 – 171) |
| Introns/transcript (range) | 6.16 (SD 5.93) (1 – 170) |
| “spliced” UTRs/transcript (range) | 2.36 (SD 0.97) (1 - 9) |
| Avg. length of introns (range) | 981.37 (SD 4,537.66) (21 – 205,601) |
| Avg. length of mono-exonic genes | 508.21 (SD 580.75) |
| Avg. length of exons (excludes mono-exonic genes) | 238.78 (SD 293.16) |
| Avg. length of first exons | 230.71 (SD 331.44) |
| Avg. length of internal exons | 240.69 (SD 271.38) |
| Avg. length of terminal exons | 236.97 (SD 354.18) |
| Avg. length of CDS (range) | 1,506.93 (SD 1,663) (150 – 52,584) |
| Avg. length of UTRs (range) | 290.49 (SD 388.87) (1 – 5,053) |
| Avg. length of primary transcripts | 7,633.07 (SD 16,698.90) |
| G+C content exonic (mono-exonic genes) | 39.96% (SD 13.11) |
| G+C content exonic (excludes mono-exonic genes) | 37.35% (SD 9.01) |
| G+C content exonic (first exons) | 37.46% (SD 11.22) |
| G+C content exonic (internal exons) | 38.50% (SD 9.97) |
| G+C content exonic (terminal exons) | 37.55% (SD 11.15) |
| G+C content intronic | 19.44% (SD 9.98) |
| G+C content UTRs | 26,95% (SD 10.69) |
